# Polymeric materials that convert local fleeting signals into global macroscopic responses†
†Electronic supplementary information (ESI) available: Synthetic procedures, characterization data, experimental details, supporting figures, and tables of data. See DOI: 10.1039/c5sc00701a



**DOI:** 10.1039/c5sc00701a

**Published:** 2015-04-09

**Authors:** Hyungwoo Kim, Matthew S. Baker, Scott T. Phillips

**Affiliations:** a Department of Chemistry , The Pennsylvania State University , 104 Chemistry Building , University Park , PA 16802 , USA . Email: sphillips@psu.edu ; Fax: +1 814 865 5235 ; Tel: +1 814 867 2502

## Abstract

We report a general design strategy for creating polymeric materials that are capable of providing global, macroscopic changes in their properties in response to specific local and fleeting stimuli. In a proof-of-concept demonstration, a single polymer is used, yet it enables selective responses to specific stimuli, and then internally drives a macroscopic change in the material (even in locations not exposed to the stimulus), where the magnitude of change is independent of the intensity of the applied stimulus.

## Abstract 2

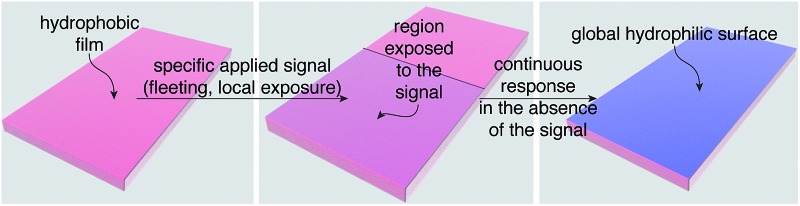
Polymers that support self-propagating reactions are used to create materials that change global wetting properties in response to specific fleeting, local stimuli.

## Introduction

**Fig. 1:**
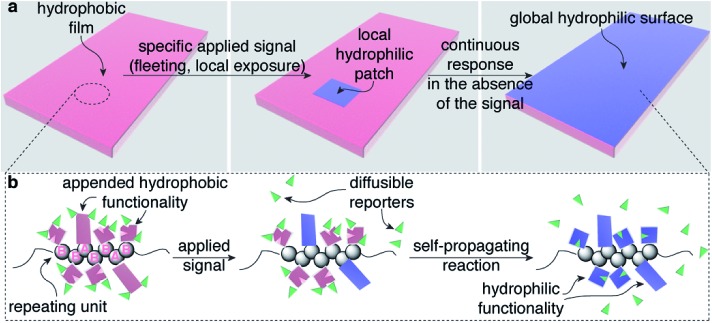
Schematic illustration of a synthetic polymeric material that is capable of changing physical properties globally in response to specific applied signals that are fleeting and/or interact with only one portion of the material. (a) A hydrophobic film that responds to a fleeting local signal (generating the blue hydrophilic region) and then converts entirely from hydrophobic (pink) to hydrophilic (blue) *via* self-propagating reactions that continue even in the absence of the signal. (b) The material is made from a random poly(norbornene) AB copolymer that contains repeating units (spheres) that are functionalized either with detection functionality (rectangles attached to repeating unit A) or functionality that mediates the self-propagating reaction (squares with missing wedges attached to repeating unit B). The green wedges represent the chemical reporters, which initially are covalently attached to the polymer, but subsequently are freed to diffuse and react with the functionality that mediates the self-propagating reaction.

The leaves of Venus flytraps and *Mimosa pudica* (touch-me-nots) close when mechanically stimulated. This macroscopic change in the structure of the leaves is controlled by biological materials within the leaves that are capable of translating a local, often fleeting signal (*e.g.*, brief touch) in one section of a leaf into a response in the entire leaf.^1–4^ Here we describe a general design strategy for creating polymeric materials that also display the ability to translate local, fleeting stimuli into global macroscopic changes in the properties of the entire material. In a proof-of-concept demonstration, we illustrate this capability using a material that switches from hydrophobic to hydrophilic (Fig. 1a), but the design strategy should be compatible with a variety of other macroscopic responses by simply mixing and matching functionality on the polymer that makes up the material.

Global, autonomous changes in the properties of these materials occur *via* self-propagating reactions within the materials that are mediated by specific functionality on each repeating unit of the polymers that make up the materials. These self-propagating reactions simultaneously communicate a detection event to distant portions of a material while also changing the properties of the material, all without requiring electronics, reagents from the surroundings, input from a user, or tethering to a specific location. In comparison, traditional synthetic stimuli-responsive materials have no mechanism for altering a distant portion of a material as a result of a local detection event.^5–8^ Thus, this work provides the foundation for a new class of bio-inspired stimuli-responsive materials.^9,10^ This unique capability, once developed further, should enable a new generation of plastics, coatings, adhesives, and other materials that autonomously reconfigure themselves in response to low intensity, limited duration exposure of a specific applied stimulus, even when the stimulus interacts with only a section of the material.

## Results and discussion

### Design strategy

Our design for these bio-inspired materials requires only a single, functionalized copolymer (gray spheres in Fig. 1b), where the AB repeating units in the polymer are distributed randomly in a 1 : 2 ratio, respectively, throughout the polymer. The functionality on the polymer includes: (i) sensing groups (pink rectangles on repeating unit A in Fig. 1b), (ii) functionality that mediates a self-propagating signal amplification reaction (pink squares with a wedge removed on repeating unit B), and (iii) polymer-bound chemical reporters (green wedges). The less abundant sensing functionality on repeating unit A responds selectively to an applied signal by undergoing a chemical reaction that changes its properties (in this case, becoming hydrophilic, Fig. 1a), while also communicating the occurrence of the detection event to other portions of the material by releasing the chemical reporters (green wedges). The functionality on repeating unit B responds to the chemical reporter by simultaneously releasing more copies of the reporter while also transforming the molecular structure attached to repeating unit B (again by becoming hydrophilic, Fig. 1a). Thus, in theory, a single detection event can trigger a sequence of reactions that transform the physical properties of the material as a whole, in this case, rendering the material hydrophilic.

### An example of a polymer that supports self-propagating reactions in response to fleeting stimuli

**Fig. 2:**
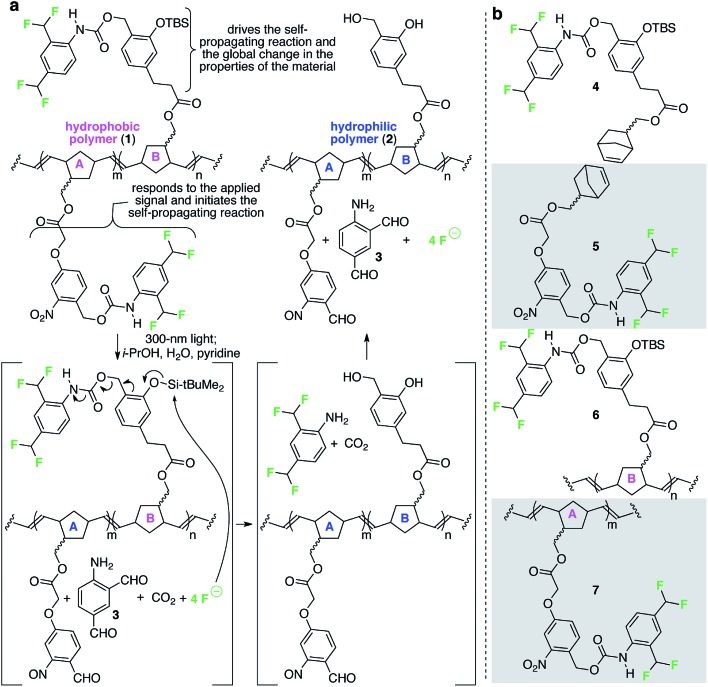
A specific polymer for creating materials that respond globally to local, fleeting applied signals. (a) This random poly(norbornene) AB copolymer is designed to respond to 300 nm light as the fleeting and local applied signal. The colors in the image match the color-coding in Fig. 1 to enable comparisons of macroscopic and molecular details of the design. (b) Structures of additional monomers and polymers described in this study. TBS = *tert*-butyldimethylsilyl.

Fig. 2 depicts a specific poly(norbornene) (**1**) that we designed to demonstrate the overall concept of materials that change globally in response to local and fleeting stimuli. In this example, the detection functionality is an *o*-nitrobenzyl carbamate, which reacts when exposed to 300 nm light (the applied signal) to release a pendant carbamate and ultimately four molecules of fluoride (the chemical reporters). The released fluoride is free to diffuse across the film to propagate the change in wetting properties by reacting with pendant *tert*-butyldimethyl silyl groups (TBS groups) on repeating units B, breaking the Si–O bond, which ultimately leads to release of four additional molecules of fluoride.^11,12^ Upon completion of these reactions, the formerly hydrophobic polymer has lost significant contributors to its hydrophobicity (*e.g.*, fluorine atoms, aromatic rings, and TBS groups) and now possesses alcohols. Consequently, the resulting polymer is significantly more hydrophilic than the starting polymer.

We used fluoride as the chemical reporter in this example because we need a signal (i) that accurately transfers the message from the detection event to initiation of the signal amplification reaction; (ii) readily diffuses across macroscopic distances along (and perhaps within) the material; (iii) is sufficiently stable and non-volatile to prevent its loss; and (iv) is sufficiently reactive with the TBS group on repeating unit B to initiate the self-propagating reaction on a reasonable timescale. We chose the TBS group on repeating unit B to receive the signal from the chemical reporter because of the high selectivity of the TBS group for fluoride-induced heterolytic cleavage of Si–O bonds, thus minimizing the possibility for spurious initiation of the self-propagating reaction in the absence of the applied signal. Finally, we used light as the applied signal for this demonstration because it is a convenient signal for providing spatiotemporal control for proof of concept illustration of local and fleeting signals.

### Characterization of a self-propagating response in a polymeric material

Prior to studying the polymer, we tested whether monomer **4** (Fig. 2b) was capable of supporting a self-propagating autoinductive reaction when exposed to substoichiometric quantities of fluoride. Thus, we treated **4** (1 mM in 10 : 4 : 1 MeCN–H_2_O–pyridine) with substoichiometric fluoride and quantified the time-dependent disappearance of **4** using liquid chromatography coupled to a mass spectrometer (LCMS). This experiment (ESI Fig. 1†) revealed that (i) all of **4** is consumed when exposed even to 0.05 equiv. fluoride; (ii) the rate of consumption of **4** is faster when exposed to quantities of fluoride that are higher than 0.05 equiv.; and (iii) the kinetics are sigmoidal. These three observations are consistent with self-propagating autoinductive reactions.^11^ Moreover, the expected amino dialdehyde product of the reaction (**3**, Fig. 2a) was observed in the LCMS chromatograms (ESI Fig. 2†), thus supporting the mechanism for conversion of **4** to the expected products outlined in Fig. 2.

Ring-opening metathesis polymerization of **4** provided homopolymer **6** (Fig. 2b), which was obtained in 92% yield with a number average molecular weight (*M*_n_) of 290 kDa and a polydispersity index (PDI) value of 1.2. Spin casting of a 5 mg mL^–1^ chloroform solution of **6** onto polypropylene provided a 4.3 nm ± 0.1 nm thick film with *x*,*z*-dimensions of 1 cm × 0.5 cm. Immersion (ESI Fig. 3a†) of 42 replicas of this film in sealed vials, each containing 0.3 mL of 100 µM fluoride in 10 : 4 : 1 i-PrOH–H_2_O–pyridine was expected to induce the autoinductive self-propagating reaction in the solid films. Several films were removed from the solution and dried at various intervals after initial exposure to fluoride (six films per interval) and their propensity to wet with water was measured. As expected, the contact angle measurements decreased sigmoidally for films with increasing durations of exposure to fluoride: global contact angles changed from 90.8° ± 0.4° to 74.9° ± 2.0° (ESI Fig. 3b†) (the error is provided at 90% confidence intervals).^13^ Atomic force microscopy (AFM) of these thin films, measured before and after exposure to different initial concentrations of fluoride, reveals statistically insignificant changes in surface roughness as a result of the self-propagating reaction: *e.g.*, 0.6 nm ± 0.2 nm surface roughness before exposure to 100 µM fluoride changing to 1.3 nm ± 0.7 nm features after 48 h of exposure (ESI Fig. 4†). Moreover, exposure of a film of **6** for 48 h to 10 : 4 : 1 i-PrOH–H_2_O–pyridine in the absence of fluoride resulted in no change in contact angle (*i.e.*, 90.8° ± 0.4° before exposure to the solvent and 89.9° ± 0.6° 48 h after exposure; the error is provided at 90% confidence intervals). Thus, the 16° decrease in contact angle after 48 h of exposure of **6** to fluoride is the consequence of changing molecular composition of the surface of the film (as designed), due to loss of fluorine atoms, aromatic rings, as well as other functionality during the self-propagating reaction (Fig. 2).

### Characterization of the response of a polymeric film to a fleeting stimulus

Taken together, these solution and solid phase experiments demonstrate the success of the self-propagating reaction as well as the ability of fluoride to act as a chemical reporter. We next evaluated the feasibility of the proposed photochemical reaction in films of homopolymer **7** (Fig. 2b), which contain only the functionality that responds to 300 nm light (the applied signal). Attenuated total reflection infrared spectroscopy (ATR-FTIR) measurements (ESI Fig. 7†) of spin-cast films of **7**, after various durations of exposure to 300 nm light, revealed complete loss of the asymmetric stretch of the carbamate from each repeating unit after 40 min of exposure. Loss of this diagnostic carbamate stretch is consistent with a successful photochemical reaction, as verified by an LCMS experiment using monomer **5**. Specifically, the photochemical reaction of **5** causes release of the pendant aniline, which ultimately releases four molecules of fluoride in subsequent azaquinone methide-mediated reactions. LCMS analysis of the products of the reaction of **5** with 300 nm light revealed the expected nitrosobenzene photochemical product as well as aminodialdehyde **3** (ESI Fig. 5 and 6†).

### Demonstration of a material that alters its macroscopic properties in response to fleeting stimuli

**Fig. 3:**
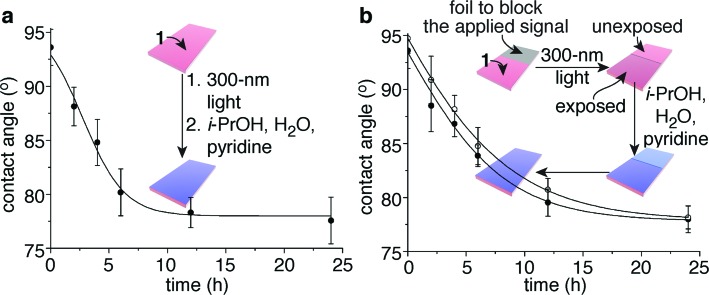
Polymeric films that completely change wetting properties in response to a local and fleeting applied signal. (a) Change in contact angle over time when a 4.0 nm ± 0.1 nm thick film of copolymer **1** was exposed to 300 nm light (the fleeting applied signal), then wet with 10 : 4 : 1 i-PrOH–H_2_O–pyridine for various durations. Each data point reflects the average value obtained from six films that were dried prior to the measurements. (b) Irradiation of one half of a film provided a local and fleeting applied signal. The open circles depict the average contact angles obtained from the unexposed half of the film, while the closed circles represent the values obtained from the exposed half (six films were measured to provide the average values for the data points). The error bars in both graphs reflect the uncertainty in the measurements at 90% confidence intervals, and the lines provide the fit of an autocatalytic equation to the data (see the ESI† for additional details).

Since homopolymers **6** and **7** individually performed successfully in their designed roles, we prepared copolymer **1**, which contains a random distribution of repeating units A and B in a 1 : 2 ratio (Fig. 2a), as confirmed by analysis of peak areas in the ^1^H NMR spectrum of the polymer (ESI Fig. 25†). This copolymer (*M*_n_ value of 160 kDa; PDI value of 1.5) readily forms films when spin cast on polypropylene. Global exposure of 36 identical films (4.0 nm ± 0.1 nm thick with *x*,*z*-dimensions of 1 cm × 0.5 cm) to 300 nm light for 40 min initiated the detection event, while immersion of each film in 0.3 mL of 10 : 4 : 1 i-PrOH–H_2_O–pyridine in a sealed container and storing them in the dark at 23 °C provided an appropriate environment for the self-propagating reaction. At various intervals, six of these films were dried and tested for their propensity to wet with water. The contact angles of the films continued to decrease over a period of ∼24 h, long after the signal (300 nm light) had been removed, for a total change of 16°. A plot of water contact angle *versus* exposure time to i-PrOH–H_2_O–pyridine reveals a temporal change in contact angle (Fig. 3a) that is commensurate with the kinetics of the self-propagating autoinductive reaction for homopolymer **6** (ESI Fig. S3†). The graph in Fig. 3a is not unambiguously sigmoidal as was observed when homopolymer **6** was treated with 100 µM fluoride (ESI Fig. S3†). However, for copolymer **1**, all of the photoresponsive groups were consumed during the 40 min irradiation with UV light (ESI Fig. S7†), therefore it is likely that considerable quantities of fluoride were generated during the photochemical reactions. High concentrations of fluoride would reduce the induction period for the autoinductive reaction, thus yielding kinetics that are comparable to the response when homopolymer **6** was treated with 1 mM or 10 mM fluoride (ESI Fig. S3†).

Control experiments support the conclusion of a signal-induced self-propagating reaction, as illustrated in ESI Fig. 8.† For example, exposure of copolymer **1** to i-PrOH–H_2_O–pyridine but not 300 nm light resulted in films with unchanged contact angles after 24 h. Likewise, exposure of homopolymer **6** to 40 min of 300 nm light followed by 24 h of wetting with i-PrOH–H_2_O–pyridine again provided statistically insignificant changes in contact angle. However, exposure of homopolymer **7** to UV light and solvent did cause a 4° initial decrease in contact angle due to the photochemical reaction, as expected, but did not continue to change further over time. Thus, the only film that provides a continuous bio-inspired response to the fleeting signal is copolymer **1**.

### Global changes in the properties of a material in response to a local stimulus

In analogy to Fig. 1a, copolymer **1** also is capable of communicating a local detection event to the entire film, thus inducing global changes in wetting properties of the film. Fig. 3b illustrates this capability, where half of the film (4.0 nm ± 0.1 nm thick with *x*,*z*-dimensions of 2 cm × 0.5 cm) was covered in foil to block the 300 nm light, while the other half was exposed to the signal. Changes in wetting properties of the unexposed half of the film tracked with the exposed half,^14^ albeit with a slight delay as expected from the bio-inspired design since the self-propagating reaction had to communicate a macroscopic change from the exposed region to the unexposed portion of the material (Fig. 3b). Hence, the films behave similarly to Venus flytraps and touch-me-nots in their ability to impart a macroscopic response to a fleeting signal.

The physical (rather than chemical) mechanism of signal propagation has yet to be established, although on going studies are probing this question. It may be possible for the fluoride to diffuse through the polymer matrix during the self-propagating reaction, particularly since the solvent may plasticize and/or swell the film. We suspect, however, that the observed changes in contact angles in this proof-of-concept study arise predominantly from diffusion of fluoride through the surrounding fluid, rather than through the polymer matrix.

## Conclusions

Overall, these results demonstrate the success of self-propagating reactions to impart synthetic materials with the ability to respond globally to signals that are both fleeting and localized. The modular design of this system should be compatible with responses to a variety of applied signals other than light by simply exchanging the detection functionality on repeating unit A (Fig. 2) with a functionality that responds to another signal, such as Pd(0),^11,15^ H_2_O_2_,^16^ enzymes,^17^ thiols,^18^ and others.^19,20^ Likewise, the chemical reporters need not be limited to fluoride: alternative privileged reporters (such as H_2_O_2_,^16^ piperidine,^20^ or thiols^18^) that have been demonstrated in small molecule autocatalytic, autoinductive, and networked reactions should be compatible with this design strategy as well. The modular design of the system also should be compatible with various structural changes to the functionality that supports the self-propagating reaction and simultaneously provides the macroscopic readout. Such changes, in theory, could be used to impart a range of macroscopic responses in materials beyond altering surface wetting properties. Finally, we anticipate that the approach will be compatible with a wide range of polymerization methods. Thus, this bioinspired strategy may provide the foundation for realizing a diverse set of synthetic materials that display response properties that extend beyond traditional stimuli-responsive materials^21–28^ and begin to resemble the behaviour of biomaterials, at least at a rudimentary level.
