# Supplementary material for: Polymeric materials that convert local fleeting signals into global macroscopic responses
Source: Chem Sci. 2015 Apr 9;6(6):3388–92. doi: 10.1039/c5sc00701a (PMC5490430; doi:10.1039/c5sc00701a)
Supplement: Supplementary file 1 [file SC-006-C5SC00701A-s001.pdf]

Electronic Supplementary Material (ESI) for Chemical Science.
This journal is © The Royal Society of Chemistry 2015

Polymeric materials that convert local fleeting signals into global macroscopic responses

Hyungwoo Kim[1], Matthew S. Baker[1], and Scott T. Phillips*

Department of Chemistry, The Pennsylvania State University, University Park, Pennsylvania 16802, United States

*Corresponding author e-mail: sphillips@psu.edu
[1]These authors contributed equally to the work.

Supporting Information

Contents

General Experimental 1
Instrumentation 2
Synthesis of Norbornene Monomers........ 5
Synthesis of Polynorbornenes........ 13
Kinetics of the Reaction of Norbornene Monomer **4** With Fluoride 15
Autoinductive Property of Homopolymer **6** 17
Kinetics Test for the Photochemical Reaction of Norbornene Monomer **5** and Homopolymer **7**........ 19
Self-propagating Response of Copolymer **1** to a Fleeting UV Signal 21
References........ 23
NMR Spectra........ 24
GPC Chromatograms 35

General Experimental

All reactions were performed in flame-dried glassware under a positive pressure of argon unless otherwise noted. All reagents used were purchased commercially and were used as received unless otherwise noted. Air- and moisture-sensitive liquids were transferred by syringe or stainless steel cannula. (4-Bromo-1,3-phenylene)dimethanol (**8**) was synthesized as described by Yeung, Schmid, and Phillips.[1] An isomeric mixture of 3-(3-

(*tert*-butyldimethylsilyloxy)-4-formylphenyl)propanoic acid (**13**) and *tert*-butyl 2-(4-(hydroxymethyl)-3-nitrophenoxy)acetate (**16**) were synthesized as described by Baker, Yadav, Sen, and Phillips.[2] Tetrahydrofuran (THF), acetonitrile (MeCN), diethyl ether, toluene (PhMe), dichloromethane (DCM), and *N*,*N*-dimethylformamide (DMF) were purified by the method developed by Pangborn et al.[3] Dry pyridine was distilled over CaH_2 at 760 mmHg. Dry isopropanol was distilled over CaH_2 at 760 mmHg and stored over 3Å molecular sieves. Deionized water was purified using a Millipore-purification system (Barnstead EASYpure® II UV/UF). Flash-column chromatography was performed as described by Still et al.,[4] employing silica gel (60-Å pore size, 32–63 µm, standard grade, Dynamic Adsorbents). Thin layer chromatography was carried out on Dynamic Adsorbents silica gel TLC (20 × 20 cm w/h, F-254, 250 µm).

Instrumentation

Proton nuclear magnetic resonance (^{1}H NMR) spectra were recorded using Bruker 300, 360 or 400 MHz NMR spectrometers at 25 °C. Proton chemical shifts are expressed in parts per million (ppm, δ scale) and are referenced to tetramethylsilane ($(CH_3)_4Si$, 0.00 ppm) or to residual protium in the solvent ($CDCl_3$, δ 7.24 ppm or CD_3OD, 3.31 and 4.78 ppm). Data are represented as follows: chemical shift, multiplicity (s = singlet, d = doublet, t = triplet, q = quartet, m = multiplet and/or multiple resonances, br = broad peak), integration, and coupling constant (*J*) in hertz. Carbon nuclear magnetic resonance (^{13}C NMR) were recorded using Bruker 300 or 400 MHz NMR spectrometers at 25 °C. Carbon chemical shifts are expressed in parts per million (ppm, δ scale) and are

referenced to the carbon resonances of the NMR solvent ($CDCl_3$, 77.23 ppm or CD_3OD, 49.15 ppm).

Gel permeation chromatography (GPC) analyses were performed using an Agilent Technologies 1200 GPC equipped with a refractive index detector, a Malvern Viscotek model 270 Dual Detector with right and low-angle light scattering, and either a Viscotek T-column (300 mm × 7.8 mm, CLM3012) and Agilent Resipore column (300 mm × 7.5 mm) in series or a single Agilent Resipore column (300 mm × 7.5 mm) using THF as the mobile phase (flow rate: 1 mL/min, 25 °C). The GPC was calibrated using monodisperse polystyrene standards from Malvern.

Liquid chromatography coupled to a mass spectrometer (LCMS) data were obtained using an Agilent Technologies 1200 series analytical reversed-phase HPLC coupled to an Agilent Technologies 6120 quadrupole mass spectrometer. The column was a BETASIL Phenyl-Hexyl column (150 mm × 2.1 mm, 5 μm particle size). The mobile phase was a mixture of 0.5 mM ammonium formate in H_2O (A), CH_3CN (B), and MeOH (C). The column was equilibrated with 3:3:4 A–B–C at a flow rate of 1 mL/min. The solvent gradient was as follows:

Time (min)	A (%)	B (%)	C (%)
0	30	30	40
15	0	60	40
17	30	30	40

A portion of the HPLC stream was automatically injected into the mass spectrometer. The mass spectrometer (APCI) settings were as follows: gas temperature of 325 °C, drying gas flow of 7 L/min, nebulizer pressure of 30 psig, and a voltage of 3000 V.

Polymer films were deposited using a Laurell WS-650MZ-23NPPB spin coater with 5 mg/mL chloroform solution at 500 rpm for 20 s followed by 2000 rpm for an

additional 20 s. One edge of each substrate was masked with Scotch tape before deposition of the polymer film. The tape was removed after deposition, and an AFM was used to measure the thickness of each film at the step created by the tape.

Contact angle measurements were performed using a Ramé-Hart automated goniometer equipped with a digital camera. Contact angles of water droplet (5 μL) on dried polymer films were analyzed using the software DROPimage Advanced. Six samples were measured at each interval, the contact angles are presented as the average of six values, and the error bars represent the deviation of the average at 90% confidence interval (90% CI).

Autocatalytic equations can be used to approximate autoinductive responses since the kinetics profiles for the two types of reactions are similar to one another. Thus, the data points (from contact angle measurements and LCMS data) were fit with the following sigmoidal Boltzmann equation[5]:

$$y = \frac{A_1 - A_2}{1 + e^{(x - x_{0})/dx}} + A_2$$

Atomic force microscopy (AFM) images were obtained at 23 °C using a Bruker Dimension Icon atomic force microscope equipped with a ScanAsyst-Air probe (triangular silicon nitride cantilevers, k = 0.4 N/m). Samples were measured in peak force tapping mode. The root mean squared roughness (R_q) was calculated from flattened images using the software Nanoscope Analysis.

Attenuated total reflection infrared spectroscopy (ATR−FTIR) measurements were performed using a Thermo Nicolet is10 instrument at rt. The spectra were obtained by averaging 32 scans over the range of 4000 cm^{-1} to 400 cm^{-1}.

Synthesis of Norbornene Monomers

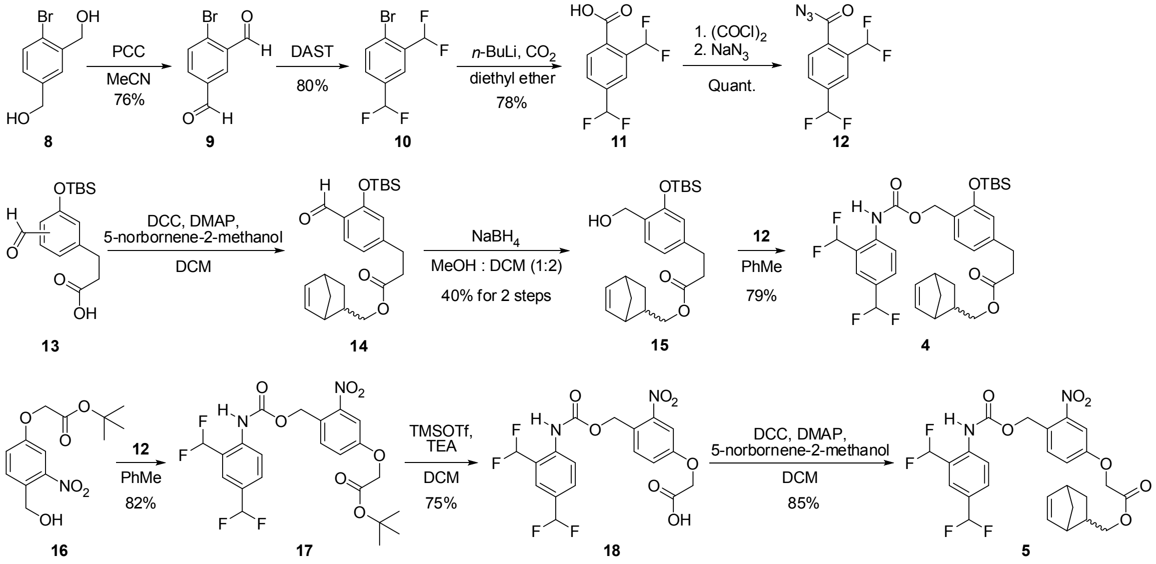

Scheme S1. Synthetic routes to norbornene monomers **4** and **5**.

4-Bromoisophthalaldehyde (9):

A suspension of compound **8**[1] (2.0 g, 9.2 mmol, 1.0 equiv) in dry MeCN was cooled to 0 °C and pyridinium dichromate (10 g, 28 mmol, 3.0 equiv) was added in portions. The reaction mixture was stirred at rt for 12 h after which the insoluble part was removed by filtering through Celite. The organic solution was concentrated under reduced pressure. The resulting residue was purified by flash column chromatography (elution with 30% EtOAc in hexanes) to provide the desired product as a white solid (1.5 g, 6.9 mmol, 76%). IR (cm^{-1}) 2955.2, 2866.8, 1683.8, 1583.1; ^{1}H NMR (360 MHz, $CDCl_3$): δ 10.46 (s, 1 H), 10.09 (s, 1 H), 8.42 (s, 1 H), 8.01 (d, 1 H, J = 7.2), 7.89 (d, 1 H, J = 7.2); ^{13}C NMR (100 MHz, $CDCl_3$): δ 190.6, 190.5, 135.8, 135.2, 134.9, 134.2, 133.9, 133.7, 133.1, 131.9, 131.7; MS (TOF MS AP–, m/z): 212.9 (M – H^+); HRMS (TOF MS EI+) Calcd. for $C_8H_5O_2Br$: 211.9473. Found: 211.9472.

1-Bromo-2,4-bis(difluoromethyl)benzene (10):

4-Bromoisophthalaldehyde **9** (1.0 g, 4.7 mmol, 1.0 equiv) was dissolved in diethylaminosulfur trifluoride (6.2 mL, 47 mmol, 10 equiv). After stirring at rt for 24 h, the resulting solution was diluted with dichloromethane (50 mL) and slow addition of water (50 mL). The organic layer was separated from the aqueous layer and washed with a saturated aqueous solution of sodium bicarbonate (3 × 50 mL). The organic solution was dried over $MgSO_4$ and concentrated under reduced pressure. The crude product was purified by flash column chromatography (elution with 10% EtOAc in hexanes) to afford the desired product as a brown oil (0.97 g, 3.8 mmol, 80%). IR (cm^{-1}) 2966.8, 1609.5, 1361.0, 1025.7; ^{1}H NMR (360 MHz, $CDCl_3$): δ 7.83 (s, 1 H), 7.77 (d, 1 H, J = 7.2), 7.54 (d, 1 H, J = 7.2), 6.96 (t, 1 H, J = 57.6), 6.70 (t, 1 H, J = 57.6); ^{13}C NMR (100 MHz, $CDCl_3$): δ 134.3, 134.1, 133.9, 133.7, 129.1, 124.8, 124.5, 124.2, 115.9, 115.1, 114.0, 113.2, 111.5, 110.5; MS (TOF MS AP–, m/z): 255.9 (M – H^+). The product did not ionize under HRMS conditions.

2,4-Bis(difluoromethyl)benzoic acid (11):

To a flame-dried round-bottom flask was added 1-bromo-2,4-bis(difluoromethyl)benzene (**10**) (1.4 g, 5.5 mmol, 1.0 equiv) and dry diethyl ether (30 mL). The resulting solution was cooled to –78 °C after which *n*-butyllithium (3.5 mL from 1.9 M in hexanes, 1.2 equiv) was added dropwise. After stirring at –78 °C for 10 min, the reaction mixture was bubbled with carbon dioxide for 15 min. The solution was stirred at rt for 50 min and then diluted with EtOAc (50 mL). The resulting solution was washed with brine (2 × 100 mL), dried over $MgSO_4$, filtered, and dried under reduced pressure. The product was

obtained as a brown solid and used without further purification (0.96 g, 4.3 mmol, 78%). IR (cm^{-1}) 2922.7, 1694.3, 1272.5, 1024.8; ^{1}H NMR (360 MHz, CD_3OD): δ 8.21 (d, 1 H, *J* = 7.2), 7.98 (s, 1 H), 7.82 (d, 1 H, *J* = 7.2), 7.64 (t, 1 H, *J* = 57.6), 6.95 (t, 1 H, *J* = 57.6); ^{13}C NMR (100 MHz, CD_3OD): δ 167.0, 138.5, 135.8, 131.5, 127.4, 122.7, 116.2, 113.9, 111.7, 109.2; MS (TOF MS AP–, m/z): 221.1 ($M - H^+$); HRMS (TOF MS ES–) Calcd. for $C_9H_5O_2F_4$ ($M - H^+$): 221.0226. Found: 221.0232.

2,4-Bis(difluoromethyl)benzoyl azide (12):

To a flame-dried round-bottom flask was added 2,4-bis(difluoromethyl)benzoic acid (**11**) (1.0 g, 4.5 mmol, 1.0 equiv) and dry dichloromethane (10 mL). The suspension was cooled to 0 °C and oxalyl chloride (0.46 mL, 5.4 mmol, 1.2 equiv) and dry DMF (35 μL, 0.45 mmol, 0.1 equiv) were added dropwise in sequence. After stirring for 1 h, the reaction mixture was concentrated under reduced pressure and the residue was re-dissolved in dry acetone (10 mL). A solution of sodium azide (0.88 g, 14 mmol, 3.0 equiv) in water (10 mL) was added dropwise at 0 °C. After stirring for 1 h, the resulting mixture was diluted with EtOAc (50 mL) and the organic layer was separated from the aqueous layer. The organic layer was washed with brine (3 × 50 mL), dried over $MgSO_4$, filtered, and concentrated. The resulting product was used for the next reaction without further purification.

Bicyclo[2.2.1]hept-5-en-2-ylmethyl 3-(3-(*tert*-butyldimethylsilyloxy)-4-formylphenyl)propanoate (14):

An isomeric mixture of compound **13**[2] (1.1 g, 3.7 mmol, 1.0 equiv) was dissolved in dichloromethane (20 mL). 5-Norbornene-2-methanol (0.66 mL, 5.5 mmol, 1.5 equiv), DMAP (90 mg, 0.73 mmol, 0.2 equiv), and *N*,*N'*-dicyclohexylcarbodiimide (1.1 g, 5.5 mmol, 1.5 equiv) were added in sequence. The reaction mixture was stirred at rt for 12 h after which insoluble precipitates were removed by filtration. The organic solution was concentrated under reduced pressure and the residue was purified by flash column chromatography (elution with 10% EtOAc in hexanes) to obtain the isomeric mixture. The resulting product was used for the next reaction without further purification.

Bicyclo[2.2.1]hept-5-en-2-ylmethyl 3-(3-(*tert*-butyldimethylsilyloxy)-4-(hydroxymethyl)phenyl)propanoate (15):

An isomeric mixture of compound **14** (1.7 g, 4.0 mmol, 1.0 equiv) was dissolved in 20 mL of dichloromethane–methanol (1:1, v/v). The solution was cooled to 0 °C and sodium borohydride (0.30 g, 8.0 mmol, 2.0 equiv) was added in portions. After stirring for 2 h, the solution was concentrated under reduced pressure. The residue was diluted with EtOAc (50 mL) and the organic solution was washed with a saturated aqueous solution of ammonium chloride (3 × 50 mL). The organic layer was dried over $MgSO_4$, filtered, and concentrated. After purifying by flash column chromatography (elution with 10% EtOAc in hexanes), the desired product was obtained as a colorless oil (0.67 g, 1.6 mmol, 40% over 2 steps). IR (cm^{-1}) 3480.9, 2985.5, 1719.8, 1526.2, 1218.6, 1151.5, 1064.0, 817.4, 742.3; ^{1}H NMR (300 MHz, $CDCl_3$): δ 7.23 (d, 1 H, J = 9.0), 6.82 (d, 1 H,

J = 9.0), 6.67 (s, 1 H), 6.09–5.91 (m, 2 H), 4.63 (s, 2 H), 4.13–3.66 (m, 2.5 H), 2.91–2.82 (m, 3.6 H), 2.62 (d, 2.4 H, *J* = 6.0), 2.04–1.87 (m, 3 H), 1.26–1.24 (m, 2.5 H), 1.03 (s, 9 H), 0.27 (s, 6 H); ^{13}C NMR (75 MHz, $CDCl_3$): δ 174.2, 154.3, 137.9, 134.1, 131.9, 130.3, 126.9, 119.0, 66.9, 65.3, 49.9, 44.5, 42.9, 35.0, 26.2, 18.9, -3.7; MS (TOF MS AP+, m/z): 434.2 (M + NH_4^+); HRMS (TOF MS ES+) Calcd. for $C_{24}H_{36}O_4SiNa$ (M + Na^+): 439.2281. Found: 439.2279.

Bicyclo[2.2.1]hept-5-en-2-ylmethyl 3-(4-((2,4-bis(difluoromethyl)phenylcarbamoyloxy)methyl)-3-(tert-butyldimethylsilyloxy)phenyl)propanoate (4):

Compound **12** (0.6 g, 2.4 mmol, 1.0 equiv) was dissolved in dry PhMe (50 mL) and stirred at 100 °C for 1 h. Compound **15** (1.0 g, 2.4 mmol, 1.0 equiv) was added, followed by additional stirring for 5 h at 100 °C. The resulting mixture was concentrated under reduced pressure and purified by flash column chromatography (elution with 10% EtOAc in hexanes) to obtain the desired product as a colorless oil (2.3 g, 3.6 mmol, 79%). IR (cm^{-1}) 3452.7, 2954.2, 1730.5, 1529.0, 1209.4, 1029.7, 838.6; ^{1}H NMR (400 MHz, $CDCl_3$): δ 8.26 (d, 1 H, *J* = 8.0), 7.63 (d, 1 H, *J* = 8.0), 7.54 (s, 1 H), 7.31 (d, 1 H, *J* = 8.0), 7.17 (s, 1 H), 6.73 (s, 1 H), 6.67 (t, 1 H, *J* = 56.0), 6.65 (t, 1 H, *J* = 56.0), 6.17–5.91 (m, 2 H), 5.22 (s, 2 H), 4.15–3.67 (m, 2 H), 2.94–2.63 (m, 6 H), 1.30 (m, 4 H), 1.03 (s, 9 H), 0.89 (m, 2 H), 0.29 (s, 6 H); ^{13}C NMR (100 MHz, $CDCl_3$): δ 172.9, 154.8, 153.3, 143.2, 137.8, 132.1, 131.1, 129.6, 124.8, 123.9, 121.3, 118.8, 115.6, 113.8, 68.0, 63.6, 49.5, 43.8, 42.4, 37.8, 35.8, 30.8, 29.0, 25.6, 18.2, -4.1; MS (TOF MS AP–, m/z): 634.4 (M – H^+); HRMS (TOF MS ES–) Calcd. for $C_{33}H_{40}NO_5F_4Si$ (M – H^+): 634.2612. Found: 634.2616.

***tert*-Butyl 2-(4-((2,4-bis(difluoromethyl)phenylcarbamoyloxy)methyl)-3-nitrophenoxy)acetate (17):**

Compound **12** (0.5 g, 2.0 mmol, 1.0 equiv) was dissolved in dry PhMe (50 mL) and stirred at 100 °C for 1 h. Compound **16**[2] (0.6 g, 2.0 mmol, 1.0 equiv) was added, followed by additional stirring for 5 h at 100 °C. The resulting mixture was concentrated under reduced pressure and purified by flash column chromatography (elution with 20% EtOAc in hexanes) to obtain the desired product as a brown solid (1.9 g, 3.7 mmol, 82%). IR (cm^{-1}) 2987.2, 1723.2, 1532.7, 1217.7, 1152.6, 1021.3, 653.9; ^{1}H NMR (360 MHz, $CDCl_3$): δ 8.23 (d, 1 H, *J* = 9.1), 7.66–7.58 (m, 4 H), 7.27–7.24 (m, 2 H), 6.74 (t, 1 H, *J* = 56.3), 6.68 (t, 1 H, *J* = 56.3), 5.60 (s, 2 H), 4.64 (s, 2 H), 1.54 (s, 9 H); ^{13}C NMR (100 MHz, $CDCl_3$): δ 167.0, 158.0, 153.0, 148.0, 137.8, 130.8, 129.1, 124.7, 124.6, 122.8, 121.9, 120.5, 118.7, 114.5, 113.9, 110.9, 83.1, 65.8, 63.8, 27.9; MS (TOF MS AP–, m/z): 501.2 ($M - H^+$); HRMS (TOF MS ES–) Calcd. for $C_{22}H_{21}N_2O_7F_4$ ($M - H^+$): 501.1285. Found: 501.1288.

2-(4-((2,4-Bis(difluoromethyl)phenylcarbamoyloxy)methyl)-3-nitrophenoxy)acetic acid (18):

A solution of compound **17** (0.50 g, 1.0 mmol, 1.0 equiv) in dry dichloromethane (20 mL) was cooled to 0 °C. Triethlyamine (1.4 mL, 10 mmol, 10 equiv) and trimethylsilyl trifluoromethanesulfonate (1.4 mL, 8.0 mmol, 8.0 equiv) were added dropwise. After stirring at rt for 6 h, the reaction mixture was washed with brine (3 × 50 mL), and the organic solution was dried over $MgSO_4$, filtered, and concentrated. The residue was

purified by flash column chromatography (elution with 10% MeOH in dichloromethane) to provide the desired product as a brown solid (0.33 g, 0.75 mmol, 75%). IR (cm^{-1}) 2990.3, 1732.7, 1604.9, 1529.9, 1222.9, 1024.5, 634.3; ^{1}H NMR (400 MHz, CD_3OD): δ 7.77 (m, 2 H), 7.70–7.65 (m, 3 H), 7.31 (d, 1 H, J = 8.0), 7.04 (t, 1 H, J = 56.0), 6.83 (t, 1 H, J = 56.0), 5.51 (s, 2 H), 4.59 (s, 2 H); ^{13}C NMR (100 MHz, CD_3OD): δ 158.6, 154.5, 148.3, 137.8, 131.7, 130.6, 130.5, 125.2, 124.0, 122.0, 120.0, 118.9, 115.7, 114.1, 112.7, 112.5, 110.7, 110.5, 63.37; MS (TOF MS AP–, m/z): 445.0 ($M – H^+$); HRMS (TOF MS ES–) Calcd. for $C_{18}H_{13}N_2O_7F_4$ ($M – H^+$): 445.0659. Found: 445.0661.

Bicyclo[2.2.1]hept-5-en-2-ylmethyl 2-(4-((2,4-bis(difluoromethyl)phenylcarbamoyloxy)methyl)-3-nitrophenoxy)acetate (5):

To a solution of compound **18** (1.0 g, 2.2 mmol, 1.0 equiv) in dichloromethane (50 mL) were added 5-norbornene-2-methanol (0.41 mL, 3.4 mmol, 1.5 equiv), 4-dimethylaminopyridine (55 mg, 0.45 mmol, 0.2 equiv), and *N*,*N*′-dicyclohexylcarbodiimide (0.70 g, 3.4 mmol, 1.5 equiv) in sequence. The reaction mixture was stirred at rt for 12 h after which insoluble precipitates were removed by filtration. The organic solution was concentrated under reduced pressure and the resulting residue was purified by flash column chromatography (elution with 20% EtOAc in hexanes) to obtain the desired product as a pale yellow solid (1.0 g, 1.9 mmol, 85%). IR (cm^{-1}) 3327.3, 2959.5, 1740.2, 1530.4, 1209.7, 1026.9, 713.7; ^{1}H NMR (360 MHz, $CDCl_3$): δ 8.23 (d, 1 H, J = 7.8), 7.69–7.58 (m, 4 H), 7.27 (m, 1 H), 6.74 (t, 1 H, J = 54.0), 6.68 (t, 1 H, J = 54.0), 6.20–5.95 (m, 2 H), 5.61 (s, 2 H), 4.75 (s, 2 H), 4.06–3.86 (m, 2 H), 2.86 (m, 2 H), 1.97–1.75 (m, 2 H), 1.48–1.13 (m, 4 H); ^{13}C NMR (100 MHz, $CDCl_3$):

δ 168.2, 161.6, 152.9, 148.9, 137.5, 132.1, 129.6, 125.2, 122.4, 120.6, 114.6, 111.1, 68.4, 67.0, 65.8, 64.1, 49.5, 45.0, 43.6, 42.2, 38.1, 28.9; MS (TOF MS AP–, m/z): 551.1 (M – H^+); HRMS (TOF MS ES–) Calcd. for $C_{26}H_{23}N_2O_7F_4$ (M – H^+): 551.1441. Found: 551.1444.

Synthesis of Polynorbornenes

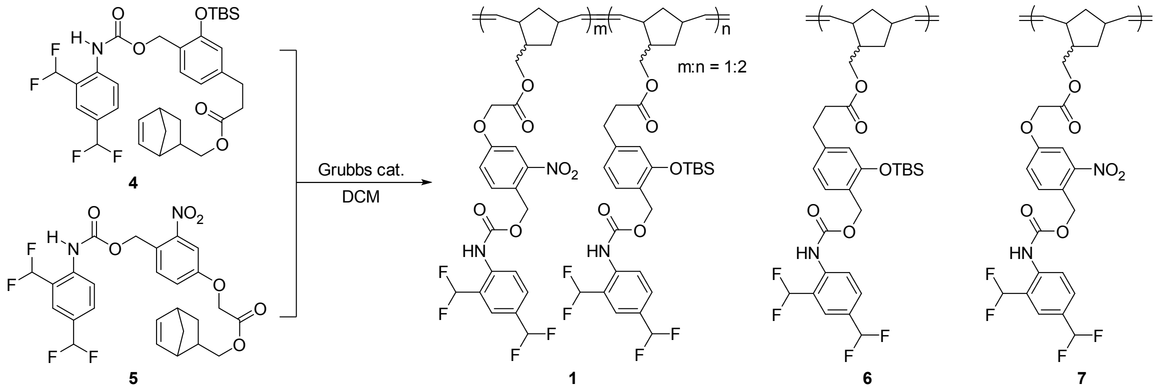

Scheme S2. Synthetic procedures for preparing copolymer **1** and homopolymers **6** and **7**.

Copolymer 1:

To a solution of compound **4** (0.30 g, 0.47 mmol, 1.0 equiv) and compound **5** (0.13 g, 0.24 mmol, 0.5 equiv) in dry dichloromethane (6 mL) was added dropwise a solution of Grubbs 2nd generation catalyst (12 mg, 14 μmol, 0.030 equiv) in dry dichloromethane (1 mL). After stirring at rt for 12 h, the reaction mixture was diluted by addition of ethyl vinyl ether (0.23 mL, 2.36 mmol, 5 equiv) and stirred for an additional 30 min. The resulting product was isolated by precipitation in MeOH. The polymer was purified by dissolving in dichloromethane and precipitating by addition of MeOH (the process was repeated twice). The desired polymer was obtained as a pale green solid (0.39 g, 90 %). Mn = 160 kDa, Mw = 240 kDa, PDI = 1.46.

Homopolymer 6:

To a solution of compound **4** (0.30 g, 0.47 mmol, 1.0 equiv) in dry dichloromethane (4 mL) was added dropwise a solution of Grubbs 2nd generation catalyst (8.0 mg, 10 μmol, 0.020 equiv) in dry dichloromethane (1 mL). After stirring at rt for 12 h, the reaction

mixture was diluted by addition of ethyl vinyl ether (0.23 mL, 2.36 mmol, 5 equiv) and stirred for an additional 0.5 h. The resulting product was isolated by precipitation in MeOH. The polymer was purified by dissolving in dichloromethane and precipitating by addition of MeOH (the process was repeated twice). The desired polymer was obtained as a pale grey solid (0.28 g, 92 %). Mn = 290 kDa, Mw = 330 kDa, PDI = 1.16.

Homopolymer 7:

To a solution of compound **5** (0.15 g, 0.23 mmol, 0.5 equiv) in dry dichloromethane (2 mL) was added dropwise a solution of Grubbs 2nd generation catalyst (3.9 mg, 4.6 µmol, 0.020 equiv) in dry dichloromethane (0.5 mL). After stirring at rt for 12 h, the reaction mixture was diluted by adding ethyl vinyl ether (0.11 mL, 1.1 mmol, 5 equiv) and stirring for an additional 0.5 h. The resulting product was isolated by precipitation in MeOH. The polymer was purified by dissolving in dichloromethane and precipitating by addition of MeOH (the process was repeated twice). The desired polymer was obtained as a pale green solid (0.14 g, 93 %). Mn = 73 kDa, Mw = 110 kDa, PDI = 1.56.

Kinetics of the Reaction of Norbornene Monomer 4 With Fluoride

The rate of consumption of monomer **4** was measured using LCMS after exposing **4** to substoichimetric quantities of fluoride. A solution of 1 mM of **4** in MeCN–H_2O–pyridine (10:4:1) was treated either with 0.5 equiv, 0.1 equiv, or 0.05 equiv of tetrabutylammonium fluoride trihydrate (the source of fluoride). Peak intensity was measured at 254 nm relative to the peak height for anisole, an internal standard.

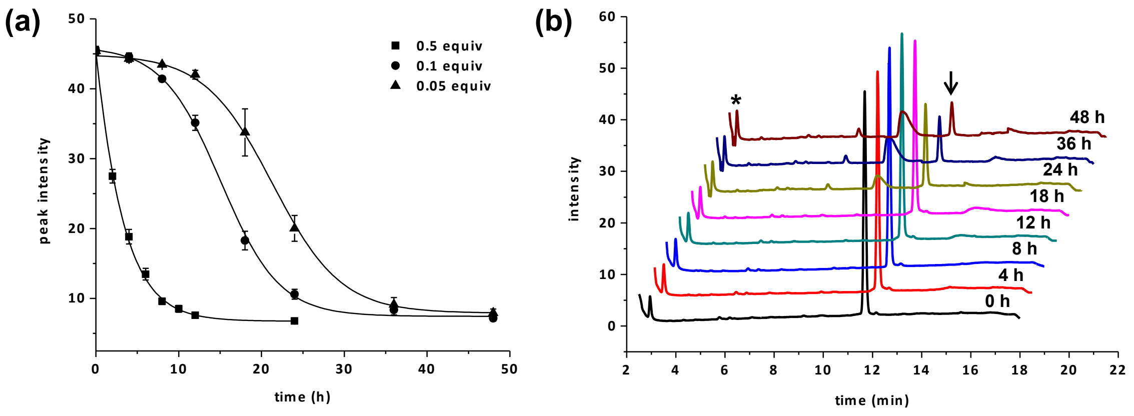

Figure S1. (a) Kinetics study for norbornene monomer **4** when exposed to 0.5 equiv of fluoride (square data points), 0.1 equiv (circle), and 0.05 equiv (triangle). The curves were obtained by fitting the data with an equation that describes autocatalytic kinetics.[5] The data points represent the averages of three measurements and the error bars reflect the standard deviations of the averages. (b) Representative overlaid LCMS spectra (254 nm) from the reaction of monomer **4** with 0.1 equiv of fluoride. Peaks at the arrow and asterisk represent monomer **4** (arrow) and anisole (asterisk). Anisole is an internal standard.

Table S1. Average peak heights that correspond to Fig. S1a.

0.5 equiv fluoride			0.1 equiv fluoride			0.05 equiv fluoride		
Time (h)	Average	Standard deviation	Time (h)	Average	Standard deviation	Time (h)	Average	Standard deviation
0	45.40	0.64	0	45.40	0.64	0	45.40	0.64
2	27.49	0.98	4	44.60	0.53	4	44.30	0.50
4	18.84	1.04	8	41.41	0.39	8	43.47	0.14
6	13.48	0.84	12	35.14	1.07	12	42.02	0.64
8	9.59	0.46	18	18.29	1.34	18	33.76	3.37
10	8.52	0.49	24	10.61	0.70	24	20.04	1.84
12	7.60	0.39	36	8.37	0.70	36	9.15	0.98
24	6.78	0.13	48	7.17	0.39	48	7.93	0.55

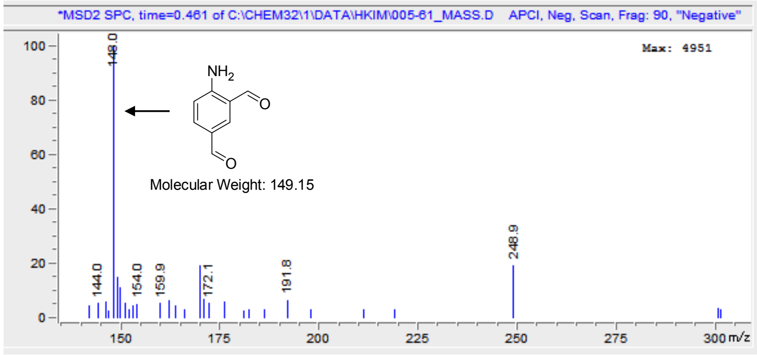

Figure S2. Representative mass spectrum (APCI, negative scan) of one peak on the LCMS chromatogram when norbornene monomer **4** is treated with 0.05 equiv of fluoride.

Autoinductive Property of Homopolymer 6

The autoinductive response of homopolymer **6** was studied using contact angle and AFM measurements. After spin-coating on a polypropylene substrate, polymer films (1 cm × 0.5 cm × 4.3 nm) were exposed to 0.3 mL of *i*-PrOH–H_2O–pyridine (10:4:1) containing 10 mM, 1 mM, 100 μM, and 0 μM (control) fluoride. Tetrabutylammonium fluoride trihydrate was used as the source of fluoride. Contact angle measurements of the polymer film were obtained at various intervals after initial exposure. This process involved removing six samples at each interval from the *i*-PrOH–H_2O–pyridine solution and drying the films by blowing air over the films and then storing the films in a vacuum oven at rt overnight. When the self-propagating reaction completed (after 48 h), films were rinsed with 1:1 MeOH–H_2O and dried using the same procedure as described above. The roughness of each surface was measured using AFM. R_q parameters were obtained from five measurements and presented as average and standard deviation of these five measurements.

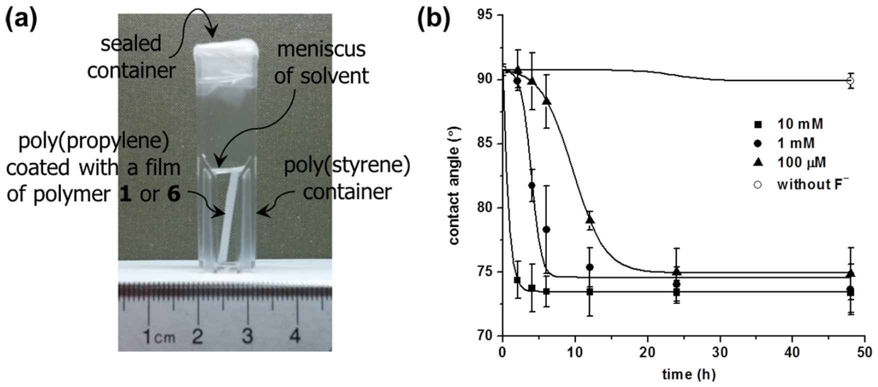

Figure S3. (a) Experimental setup for films of polymer **1** and **6** spin-cast on poly(propylene). After immersion of films in *i*-PrOH–H_2O–pyridine (10:4:1), the cuvettes were sealed with a lid and parafilm to prevent solvent evaporation. No change in solvent volume was apparent over the course of the experiments. (b) Change in contact angle over time when a polymer film of homopolymer **6** was exposed to 10 mM (square data points), 1 mM (closed circles), and 100 μM fluoride (triangles). The control was conducted without fluoride (open circles). Each data point represents the average of six measurements obtained from six different films. The error bars reflect the variation in the measurements at 90% confidence intervals.

Table S2. Contact angle data corresponding to Fig. S3. The title 90% CI reflects the variation in the measurements at 90% confidence intervals.

	10 mM fluoride		1 mM fluoride		100 μM fluoride		Without fluoride	
Time (h)	Average	90% CI	Average	90% CI	Average	90% CI	Average	90% CI
0	90.8	0.4	90.8	0.4	90.8	0.4	90.8	0.4
2	74.4	1.5	89.9	0.6	90.7	1.6		
4	73.8	1.9	81.8	1.3	89.9	2.2		
6	73.5	1.2	78.3	3.4	88.3	2.1		
12	73.5	1.9	75.4	1.5	79.0	0.7		
24	73.5	0.9	74.1	1.3	75.0	1.8		
48	73.4	1.5	73.7	2.0	74.9	2.0	89.9	0.6

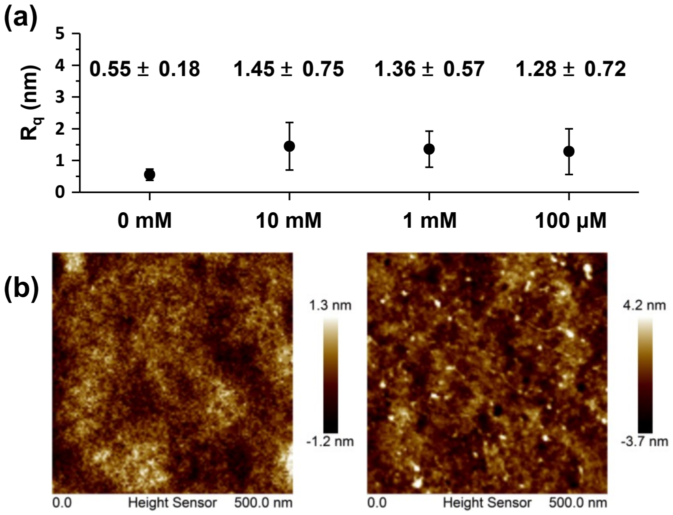

Figure S4. (a) Surface roughness (R_q) values of films of homopolymer **6** calculated after exposure to different initial quantities of fluoride for 48 h. (b) Representative AFM topographies of the films stored for 48 h in 10:4:1 *i*-PrOH–H_2O–pyridine without being exposed to fluoride (left) and after being exposed to 100 μM fluoride (right). The differences in surface roughness measurements are insignificant as determined by the Student's t-test at 95% confidence intervals.

Kinetics Test for the Photochemical Reaction of Norbornene Monomer 5 and Homopolymer 7

A solution of 1 mM of **5** in MeCN–H_2O–pyridine (10:4:1) was irradiated using a Rayonet photochemical reactor equipped with 300-nm lamps at rt. Irradiation was performed using a Rayonet RPR-100 photochemical reactor equipped with 14 RPR-3000 lamps (21 W each), which have a maximum emission intensity at 300 nm. Aliquots of the reaction mixture were analyzed by LCMS every 10 min. Peak intensity was measured at 254 nm relative to the peak height for anisole, an internal standard.

The photochemical reaction of a polymer film of homopolymer **7** was monitored by ATR-FITR measurement. Homopolymer **7** was spin-coated as a 5 mg/mL chloroform solution on a polypropylene substrate and irradiated using a Rayonet photochemical reactor equipped with 300-nm lamps at rt. The IR measurement was performed every 10 min.

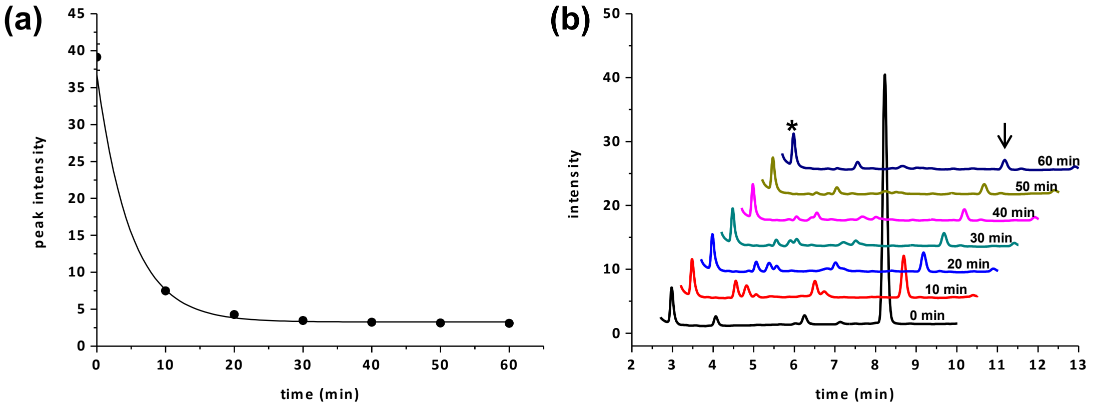

Figure S5. Kinetics study for norbornene monomer **5** when irradiated with 300-nm light. (a) The curve was obtained by fitting relative LCMS peak height data (see part b) with the Boltzmann equation. The data points represent the averages of three measurements and the error bars reflect the standard deviations of the averages. (b) Representative overlaid LCMS spectra (254 nm) from the photochemical reaction of monomer **5** with UV irradiation. Peaks at the arrow and asterisk represent monomer **5** (arrow) and anisole (asterisk). Anisole is an internal standard.

Table S3. Average peak heights that correspond to Fig. S5a.

Time (min)	Average	Standard deviation
0	39.12	1.78
10	7.48	0.12
20	4.28	0.11
30	3.45	0.07
40	3.23	0.07
50	3.13	0.10
60	3.09	0.10

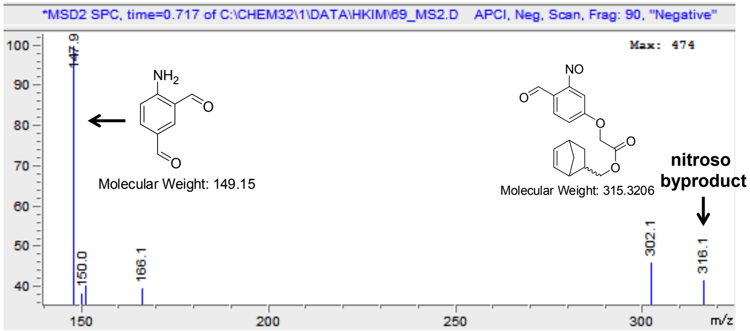

Figure S6. Mass spectrum (APCI, negative scan) of the reaction mixture when norbornene monomer **5** is irradiated with 300-nm light for 60 min.

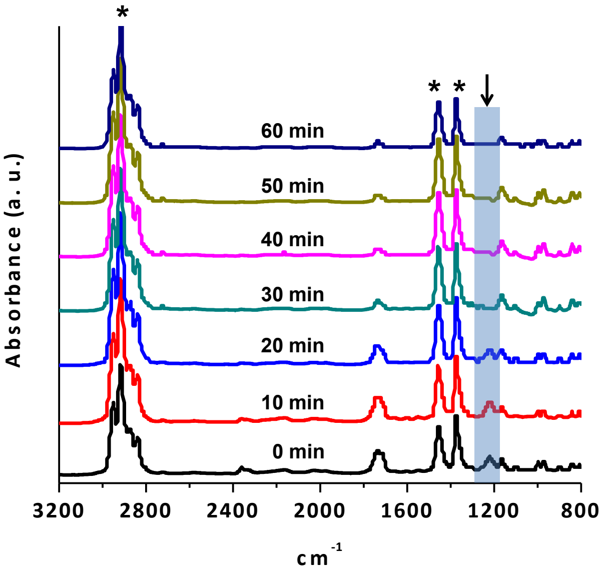

Figure S7. Overlaid ATR-FTIR spectra obtained from a film of homopolymer **7** on polypropylene substrate. The film was exposed to 300-nm light, and the IR spectra were acquired every 10 min. Peaks at the arrow and asterisks correspond to the carbamate (asymmetric stretch) in **7**, and the polypropylene substrate, respectively.

Self-propagating Response of Copolymer 1 to a Fleeting UV Signal

The self-propagating response of copolymer **1** was studied using contact angle measurements. After spin-coating **1** on a polypropylene substrate, the polymer films (1.0 cm × 0.5 cm × 4.0 nm) were irradiated in a Rayonet photochemical reactor equipped with 300-nm lamps at rt for 40 min. The irradiated samples were wet with 0.3 mL of *i*-PrOH–H_2O–pyridine (10:4:1). Contact angles of the polymer films were measured at various intervals after initial exposure to UV light. Six samples were measured at each interval after removing the sample from the solution and drying the sample by blowing air over the film and storing the film in a vacuum oven at rt overnight. Identical experiments were conducted for homopolymers **6** and **7**.

To test for a global macroscopic response, half of a film of **1** (2 cm × 0.5 cm surface area) was covered with aluminum foil prior to UV irradiation. After irradiation, the foil was removed, the film was wet with 0.6 mL of *i*-PrOH–H_2O–pyridine (10:4:1), and contact angle measurements of both halves (exposed and unexposed to UV) of the film were acquired at various intervals. At each interval, six samples were removed from the solution and dried by blowing air over the film and storing the film in a vacuum oven at rt overnight prior to measurements of contact angles.

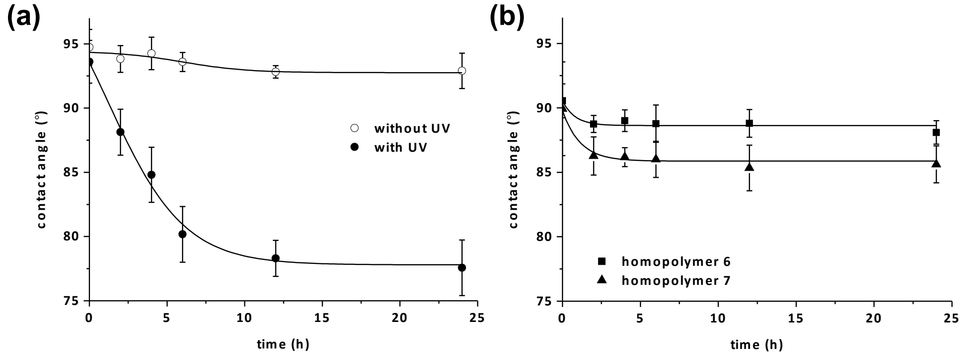

Figure S8. (a) Change in contact angle over time when a film of **1** was irradiated under 300-nm light for 40 min and wet with 10:4:1 *i*-PrOH–H_2O–pyridine (closed data points). Change in contact angle when a film of **1** was not exposed to UV light, but was exposed to 10:4:1 *i*-PrOH–H_2O–pyridine (open circles). (b) Change in contact angle over time when polymer films of **6** (square data points) and **7** (triangular data points) were irradiated with 300-nm light for 40 min and wet with 10:4:1 *i*-PrOH–H_2O–pyridine. The data points in both graphs reflect the average of six measurements, and the error bars depict the variation in the measurements at 90% confidence intervals.

Table S4. Contact angle data corresponding to Fig. S8. The label 90% CI reflects the variation in the measurements at 90% confidence intervals.

	**1** with UV		**1** without UV		**6** with UV		**7** with UV	
Time (h)	Average	90% CI	Average	90% CI	Average	90% CI	Average	90% CI
0	93.6	1. 7	94.7	1.4	90.5	1.3	89.9	3.6
2	88.1	1.8	93.8	1.0	88.7	0.7	86.3	1.5
4	84.8	2.1	94.3	1.3	89.0	0.8	86.2	0.7
6	80.2	2.2	93.6	0.7	88.8	1.4	86.0	1.4
12	78.3	1.4	92.8	0.5	88.8	1.1	85.3	1.8
24	77.6	2.2	92.9	1.4	88.1	0.9	85.6	1.4

Table S5. Contact angle data for copolymer **1** in Fig. 3b. The label 90% CI reflects the variation in the measurements at 90% confidence intervals.

	Exposed half		Unexposed half	
Time (h)	Average	90% CI	Average	90% CI
0	93.6	1.7	94.7	1.4
2	88.5	2.5	90.9	2.2
4	86.8	1.2	88.2	1.3
6	83.9	1.0	84.8	1.7
12	79.5	1.3	80.7	1.0
24	77.9	1.3	78.1	1.1

References

1. Yeung, K., Schmid, K. M. & Phillips, S. T. A thermally-stable enzyme detection assay that amplifies signal autonomously in water without assistance from biological reagents. *Chem. Commun.* **49,** 394–396 (2013).
2. Baker, M. S., Yadav, V., Sen, A. & Phillips, S. T. A self-powered polymeric material that responds autonomously and continuously to fleeting stimuli. *Angew. Chem. Int. Ed.* **52,** 10295–10299 (2013).
3. Pangborn, A. B., Giardello, M. A., Grubbs, R. H., Rosen, R. K. & Timmers, F. J. Safe and convenient procedure for solvent purification. *Organometallics* **15,** 1518–1520 (1996).
4. Still, W. C., Kahn, M. & Mitra, A. Rapid chromatographic technique for preparative separations with moderate resolution. *J. Org. Chem.* **43,** 2923–2925 (1978).
5. Nicolis, G. & Prigogine, I. Fluctuations in nonequilibrium systems. *Proc. Nat. Acad. Sci.* **68,** 2102–2107 (1971).

NMR Spectra

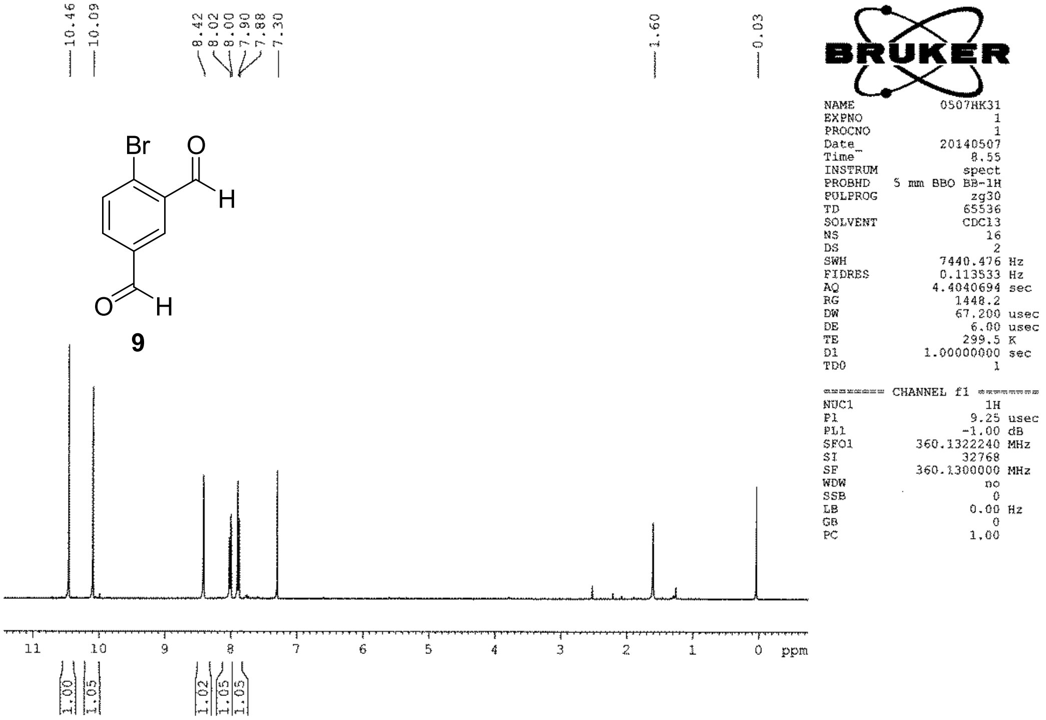

Figure S9. ^{1}H NMR spectrum of **9**.

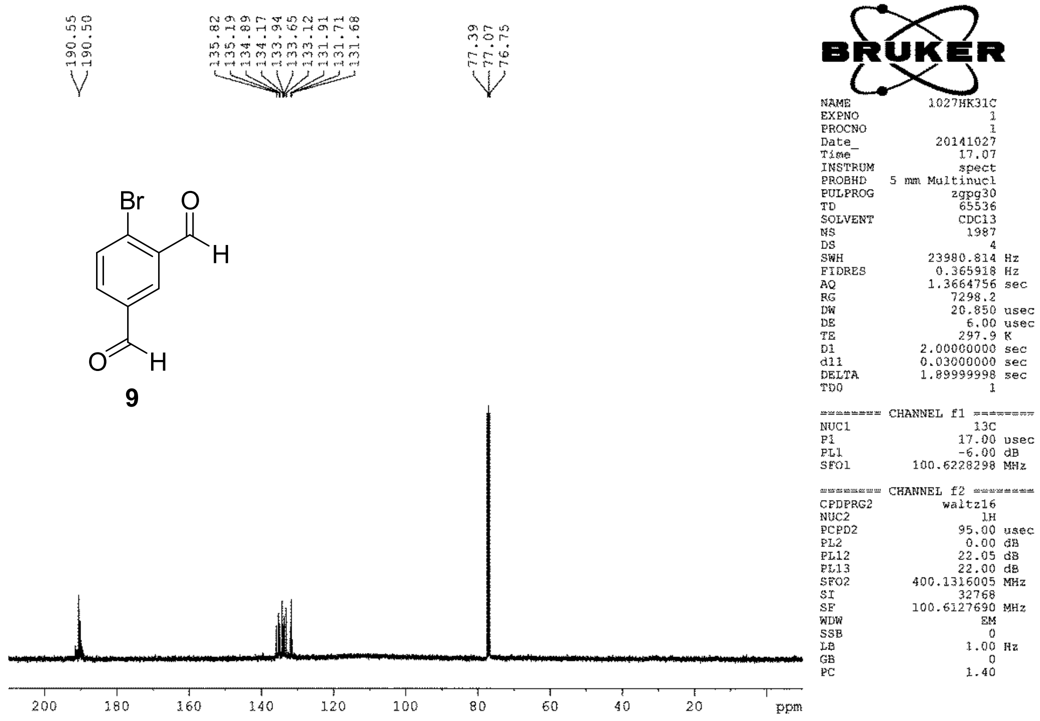

Figure S10. ^{13}C NMR spectrum of **9**.

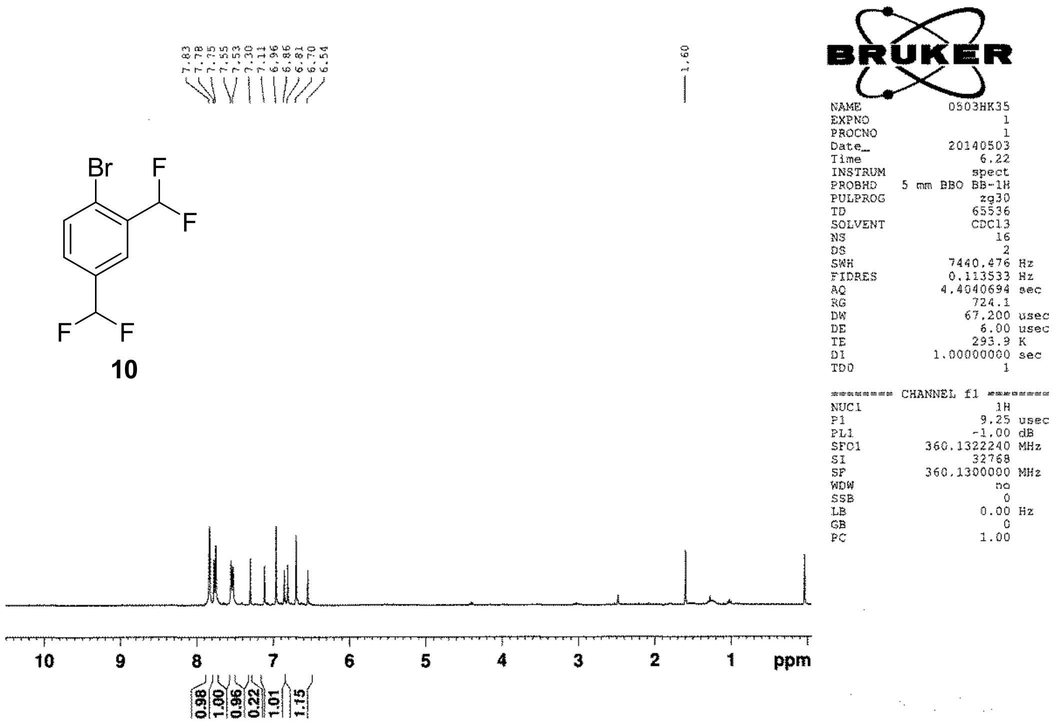

Figure S11. ^{1}H NMR spectrum of **10**.

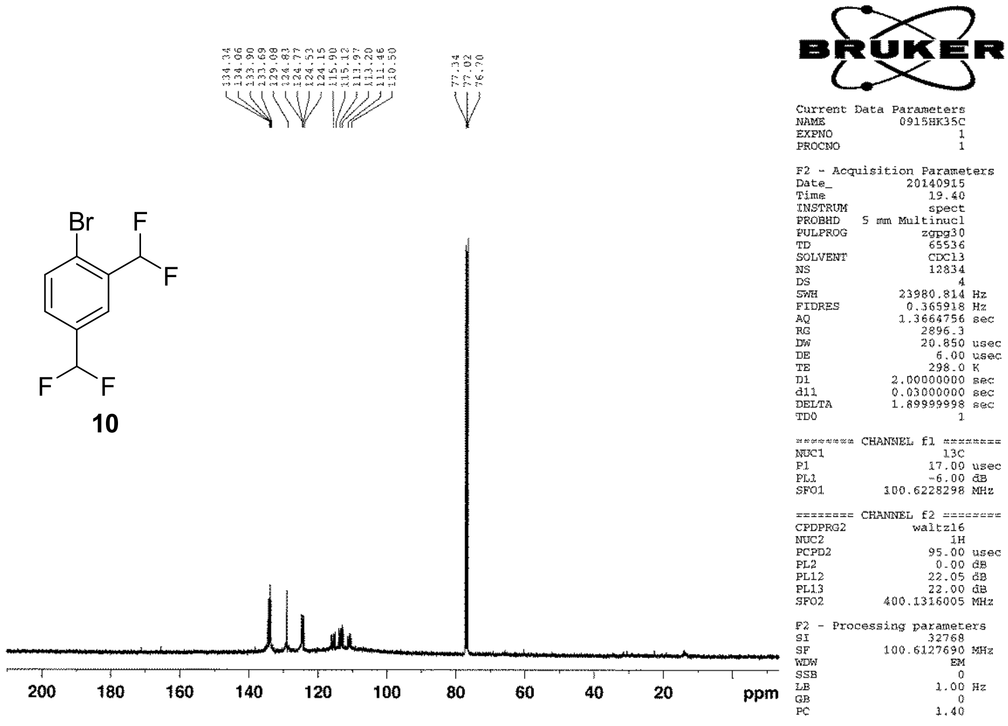

Figure S12. ^{13}C NMR spectrum of **10**.

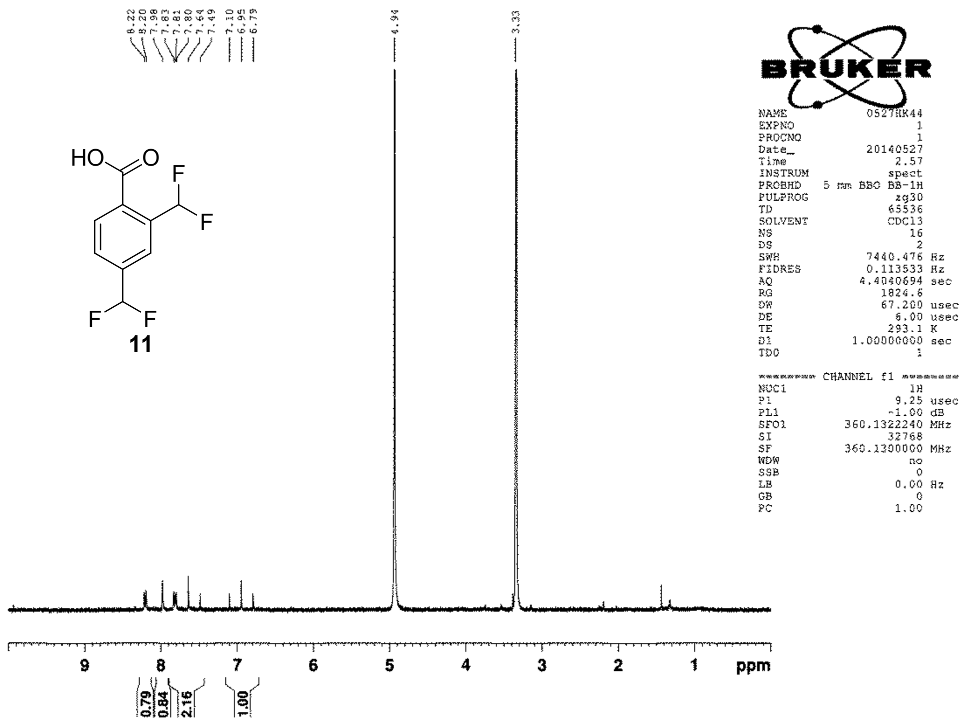

Figure S13. ^{1}H NMR spectrum of **11**.

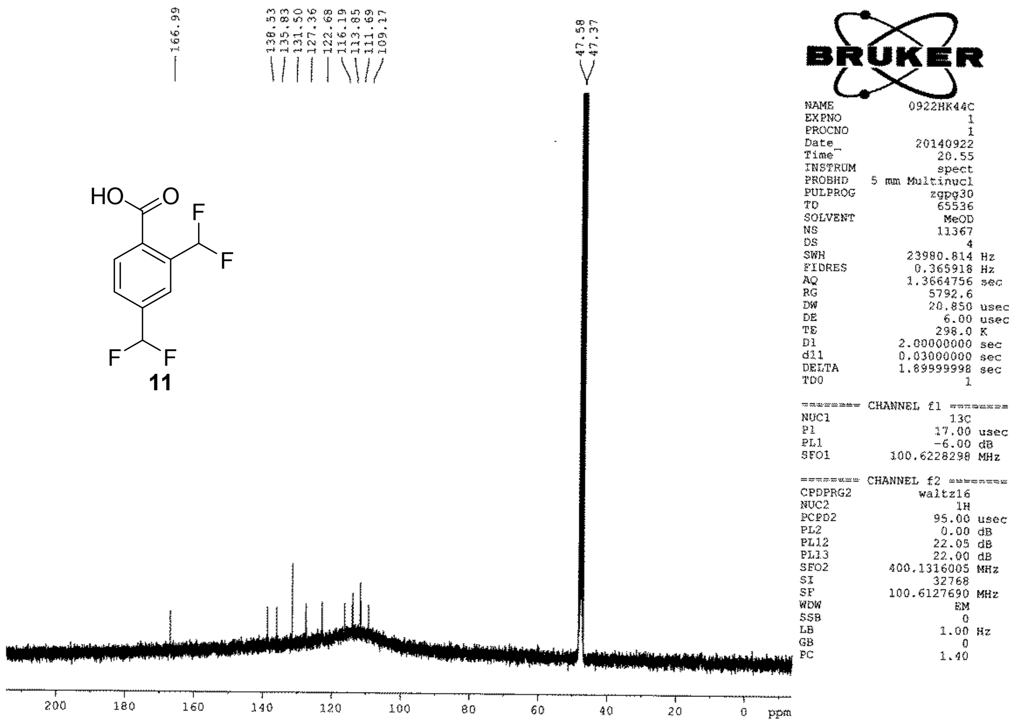

Figure S14. ^{13}C NMR spectrum of **11**.

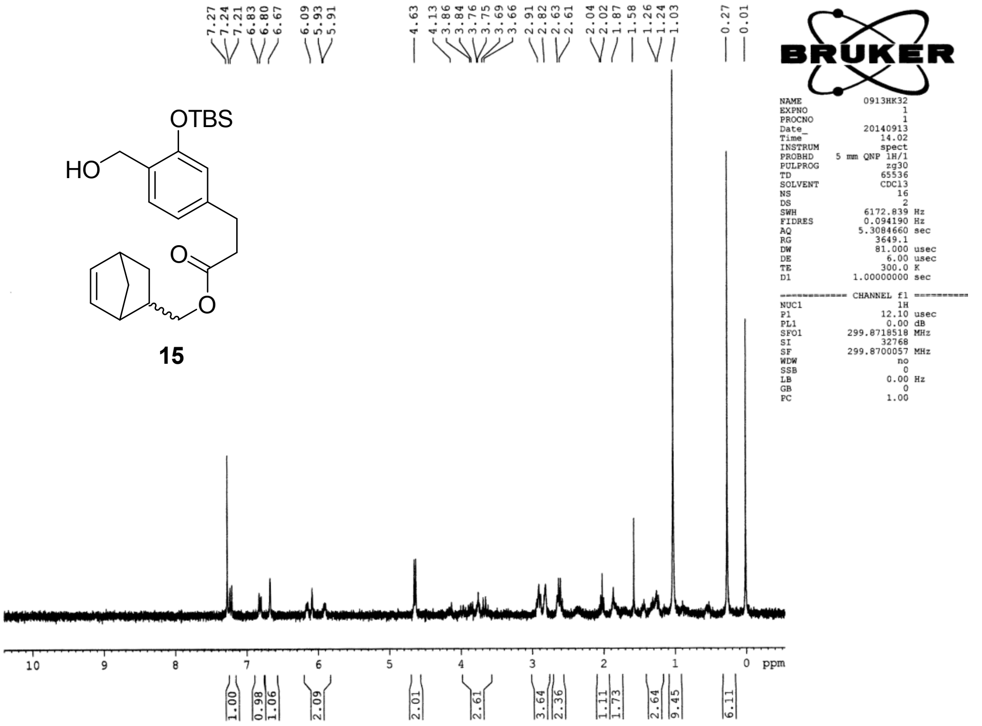

Figure S15. ^{1}H NMR spectrum of **15**.

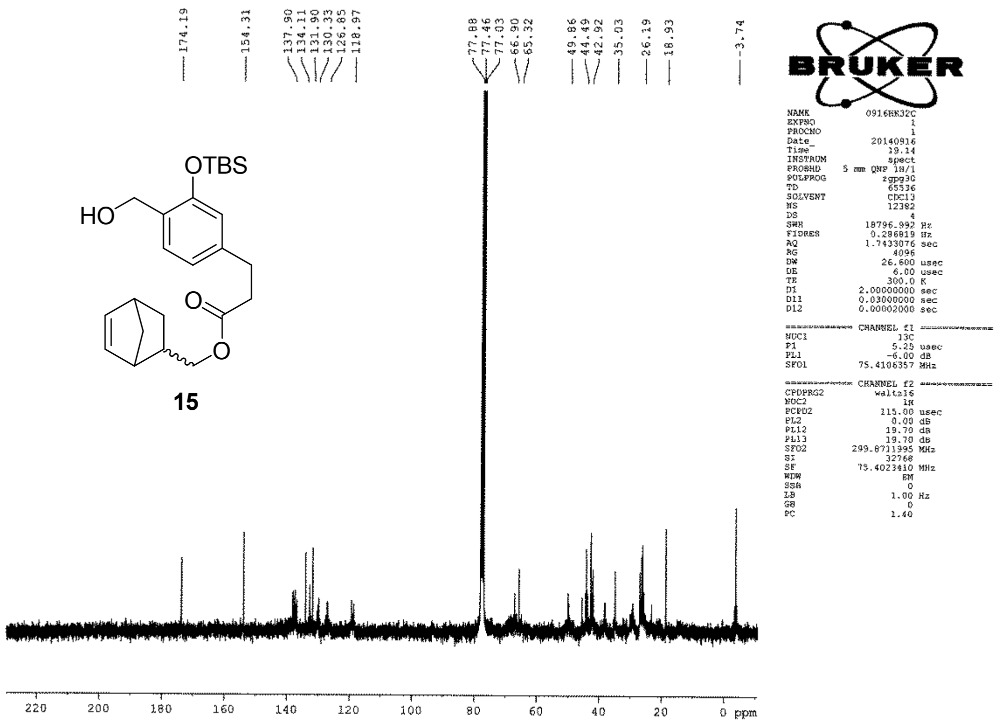

Figure S16. ^{13}C NMR spectrum of **15**.

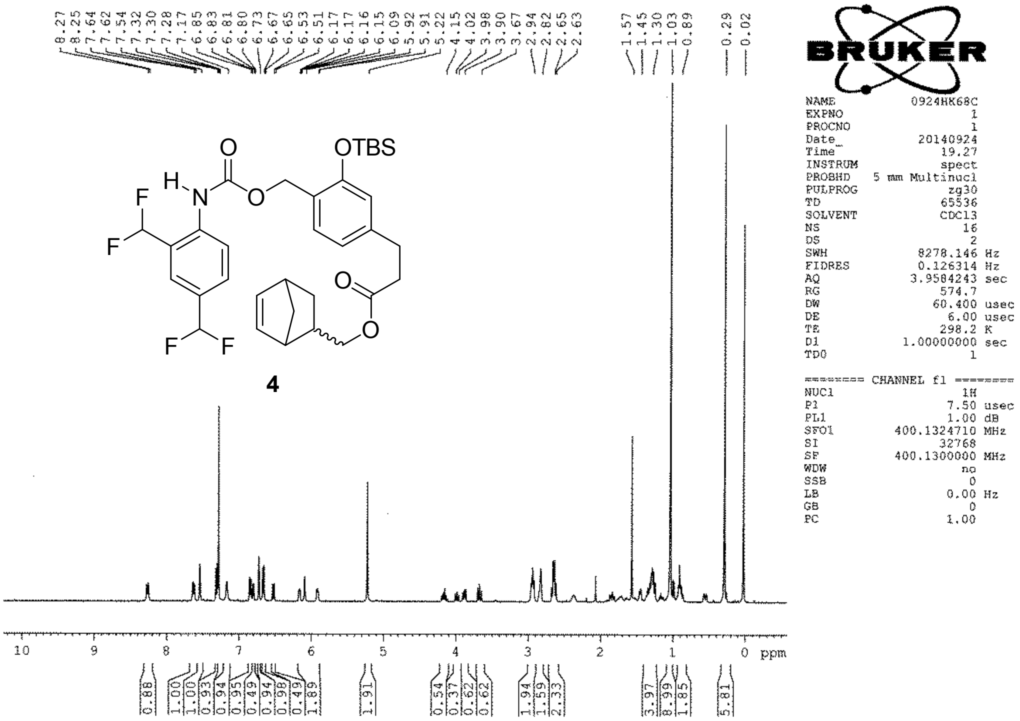

Figure S17. ^{1}H NMR spectrum of **4**.

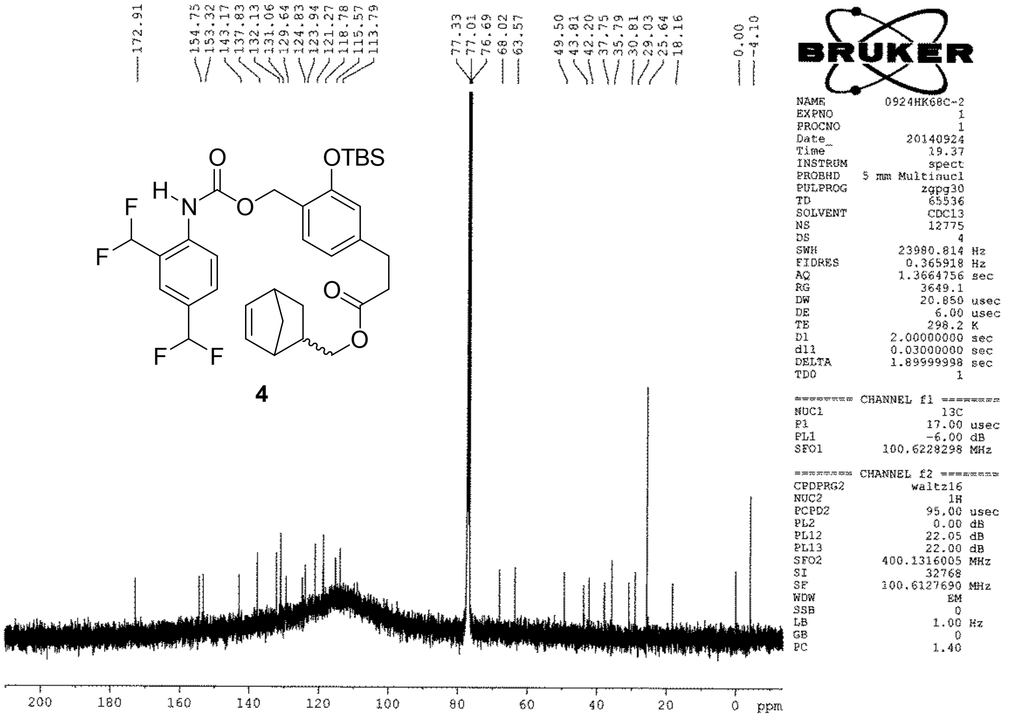

Figure S18. ^{13}C NMR spectrum of **4**.

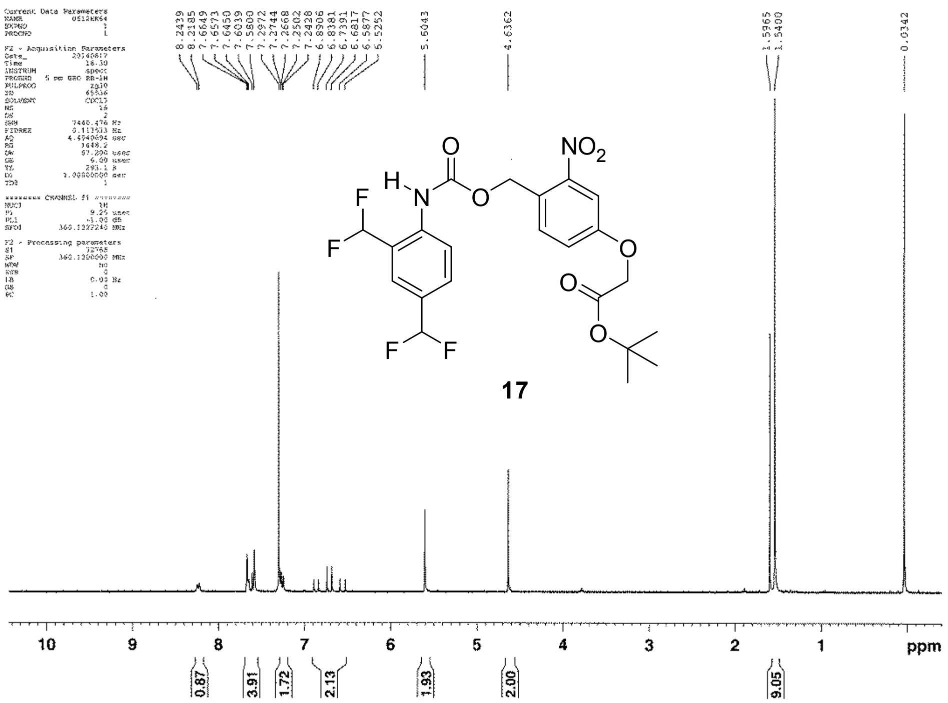

Figure S19. ^{1}H NMR spectrum of **17**.

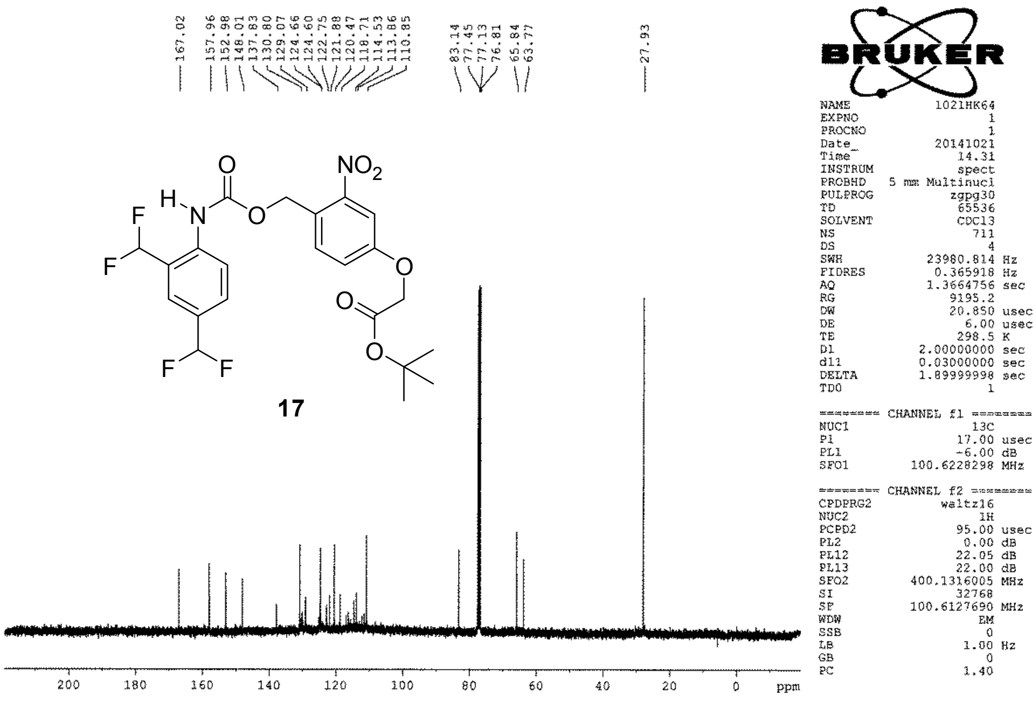

Figure S20. ^{13}C NMR spectrum of **17**.

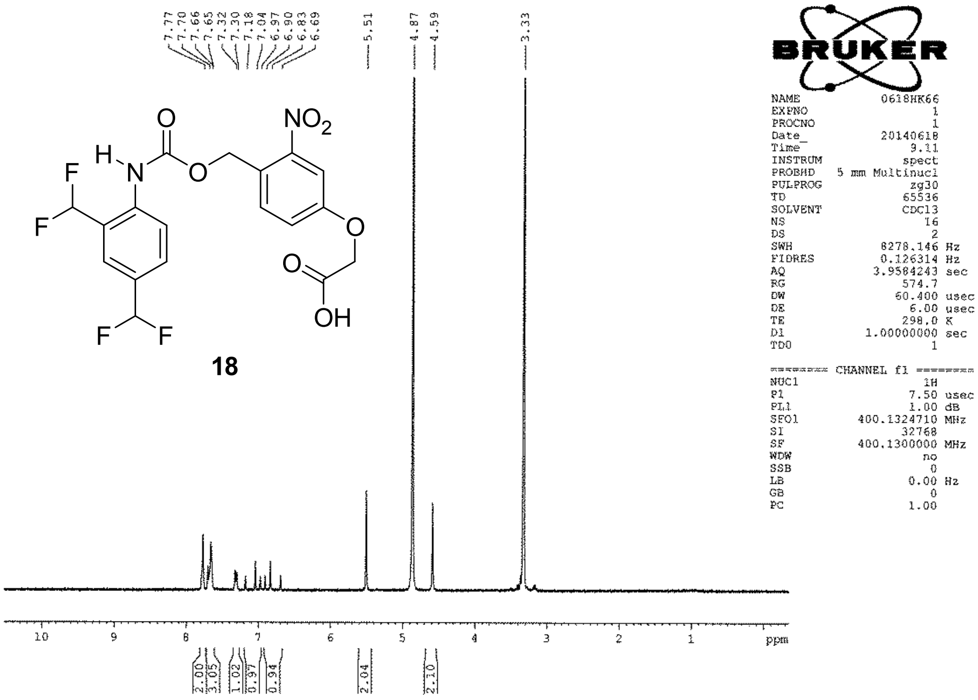

Figure S21. ^{1}H NMR spectrum of **18**.

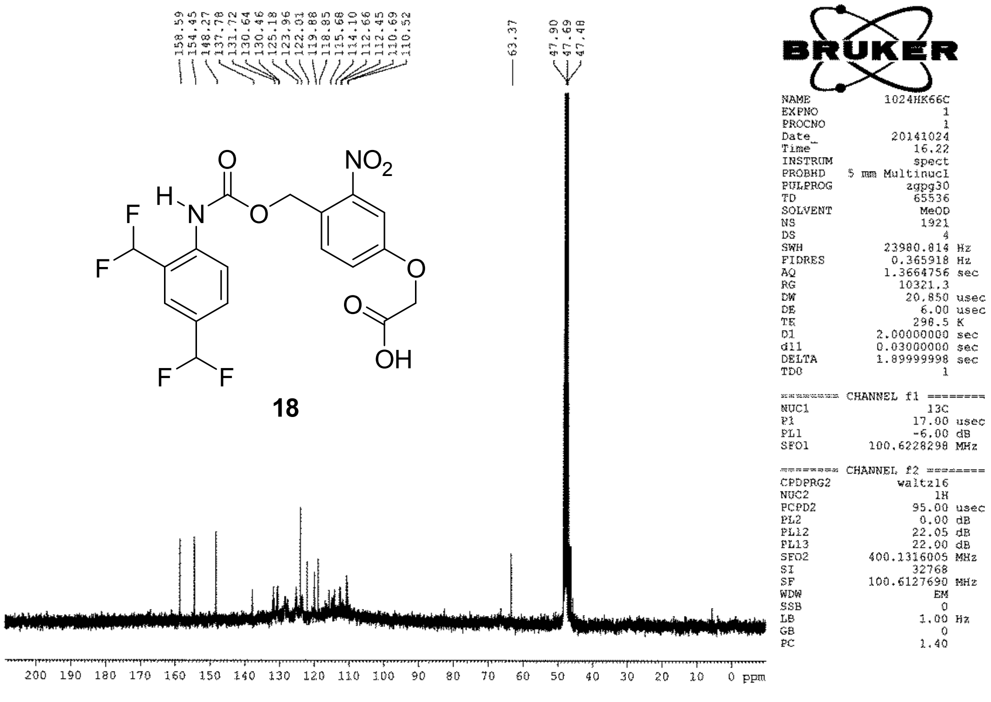

Figure S22. ^{13}C NMR spectrum of **18**.

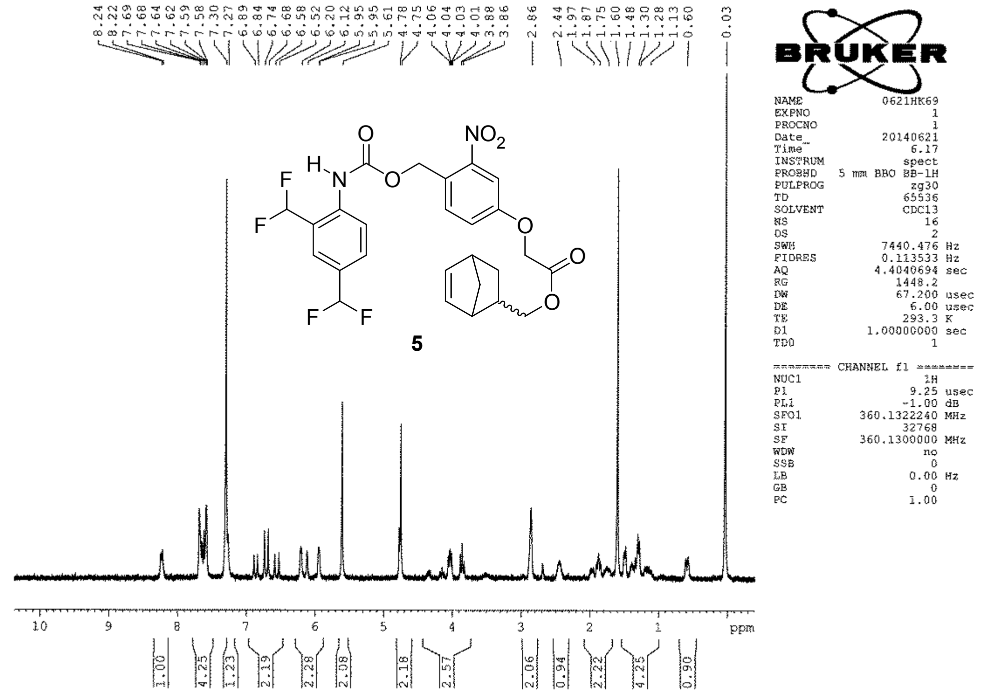

Figure S23. ^{1}H NMR spectrum of **5**.

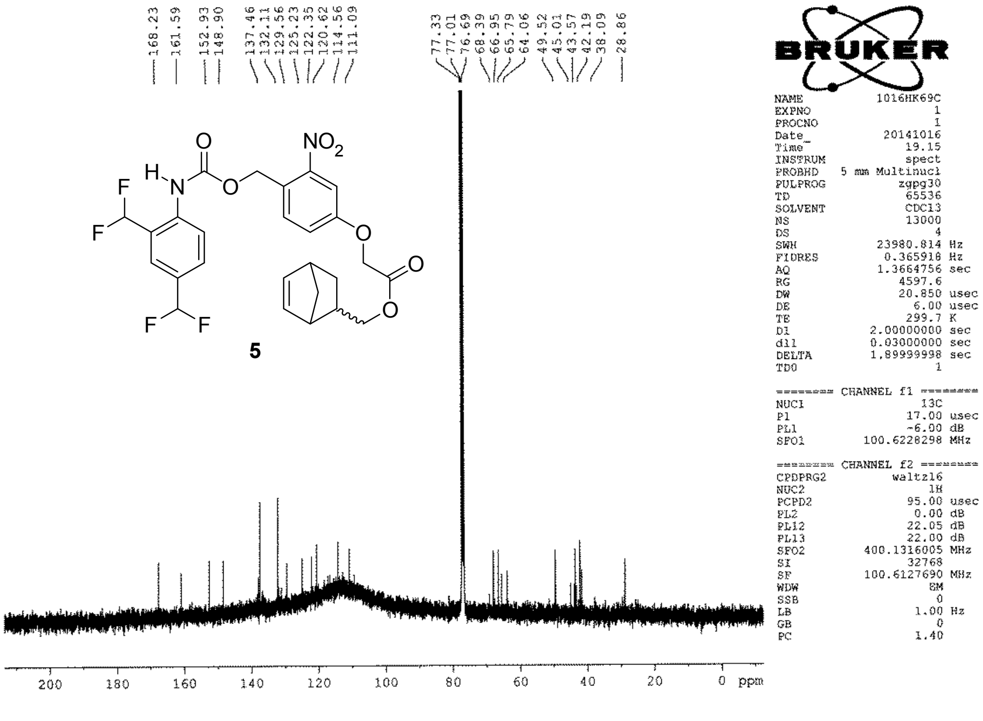

Figure S24. ^{13}C NMR spectrum of **5**.

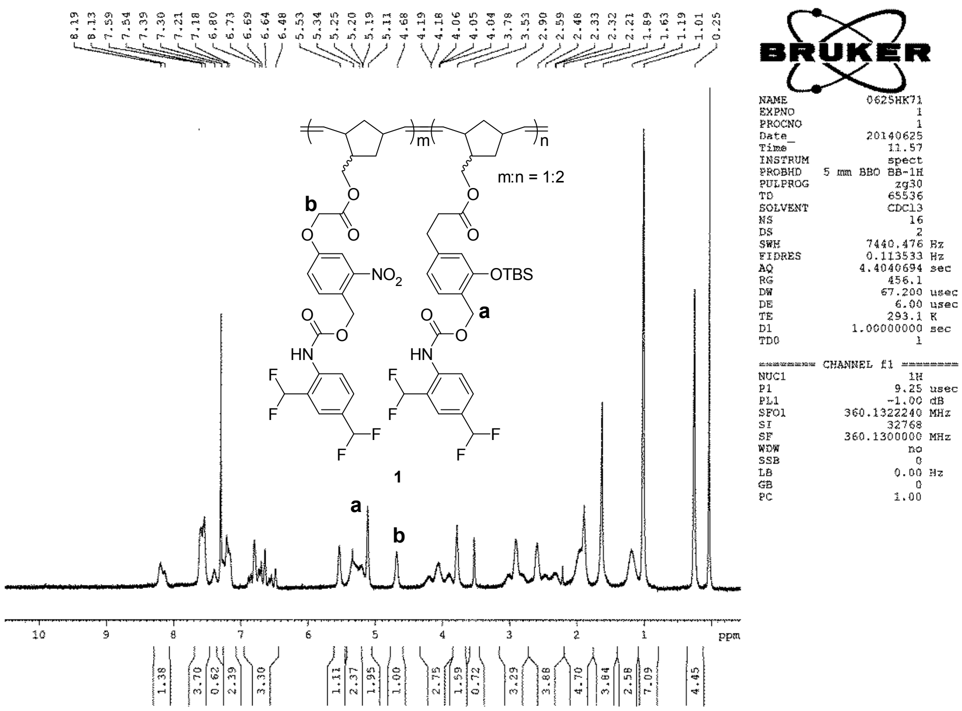

Figure S25. ^{1}H NMR spectrum of **1**. Based on the integration ratio between **a** and **b**, the monomer composition of the polymer was determined to be similar to the feeding ratio (i.e., m:n = 1:2).

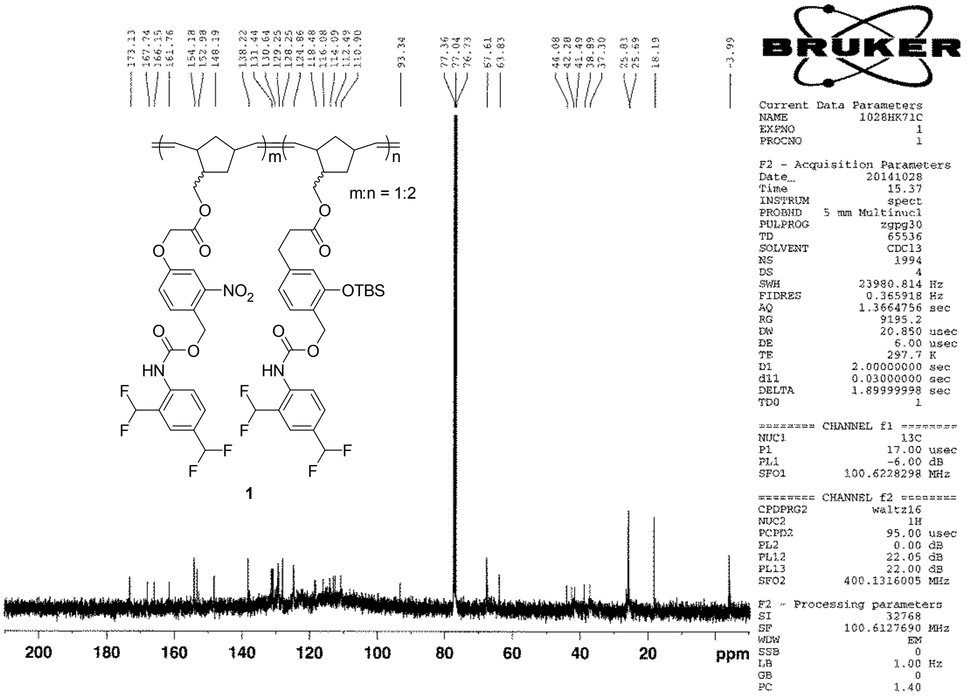

Figure S26. ^{13}C NMR spectrum of **1**.

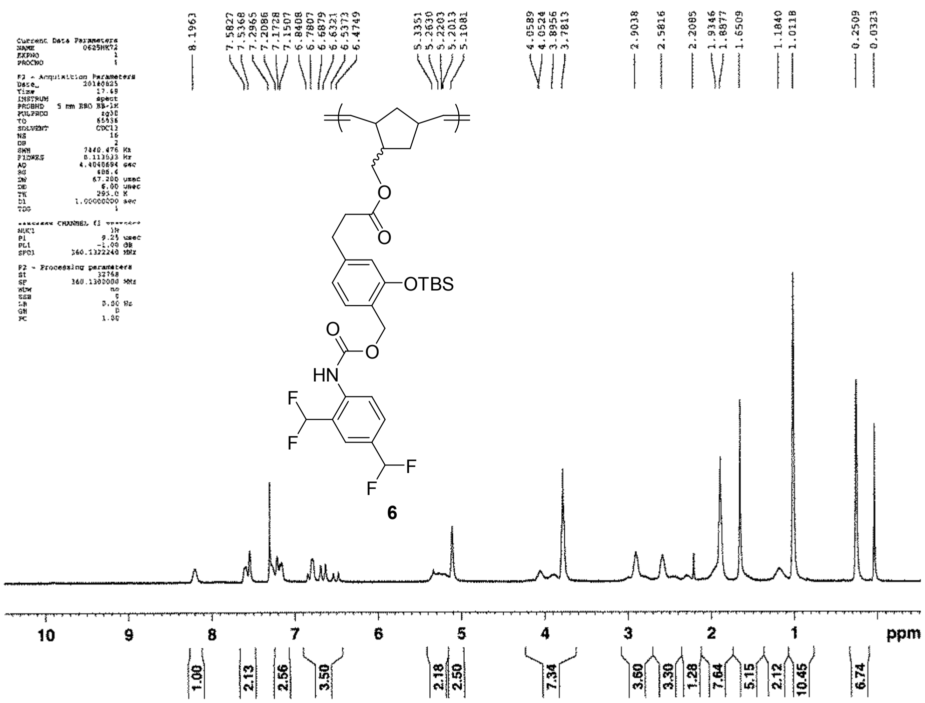

Figure S27. ^{1}H NMR spectrum of **6**.

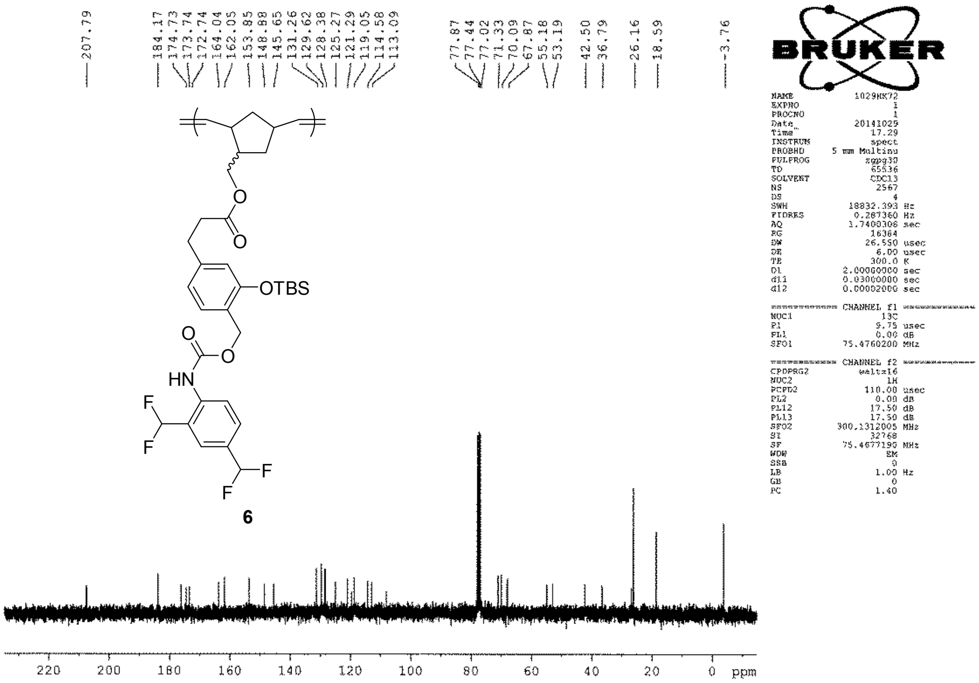

Figure S28. ^{13}C NMR spectrum of **6**.

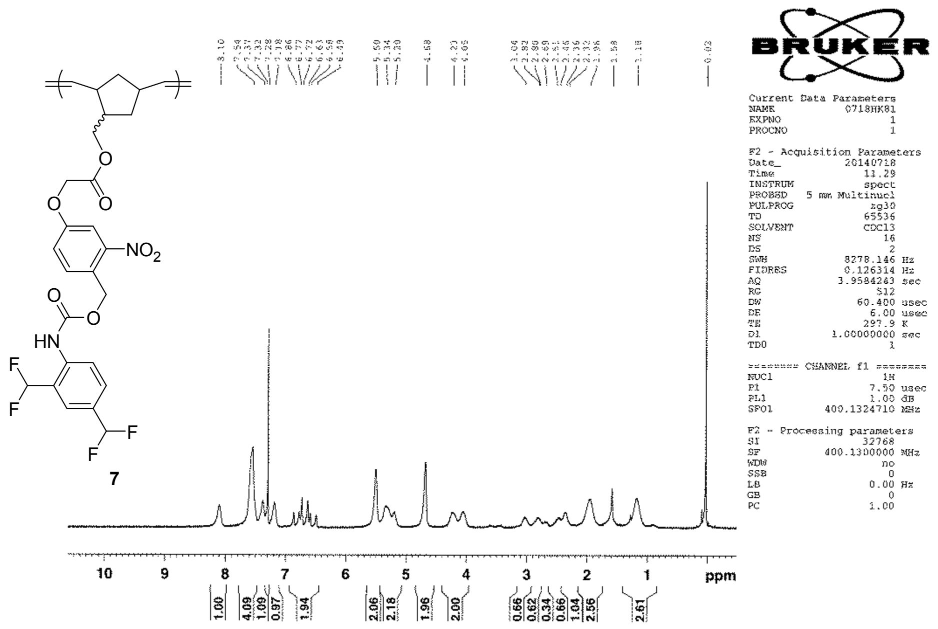

Figure S29. ^{1}H NMR spectrum of **7**.

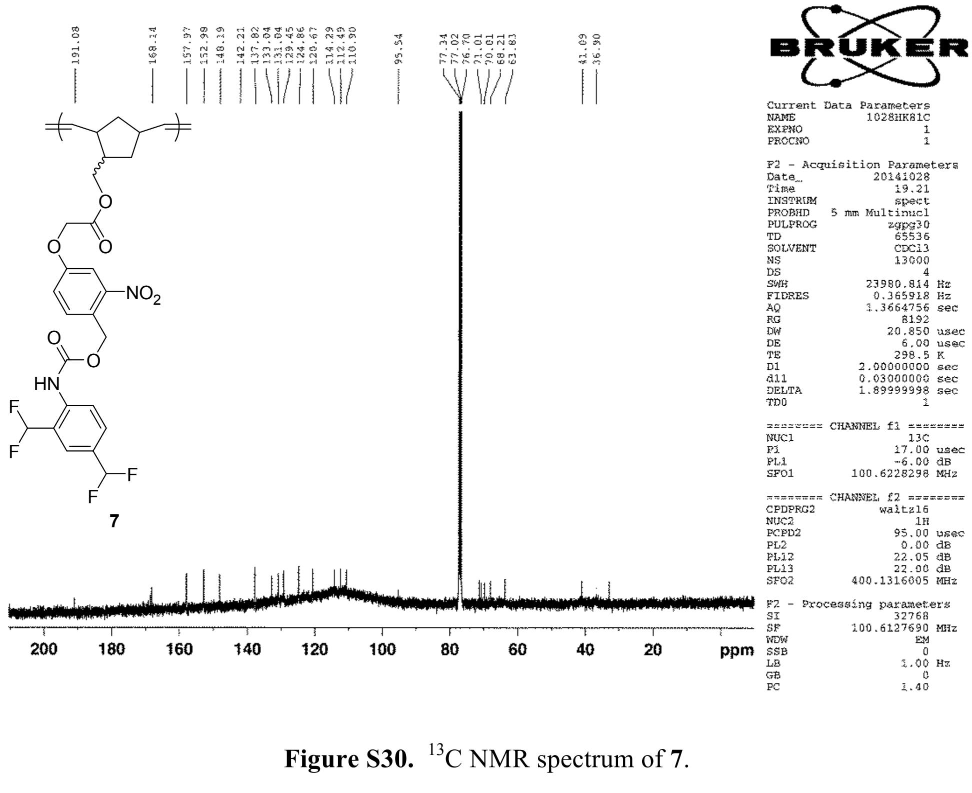

Figure S30. ^{13}C NMR spectrum of **7**.

GPC Chromatograms

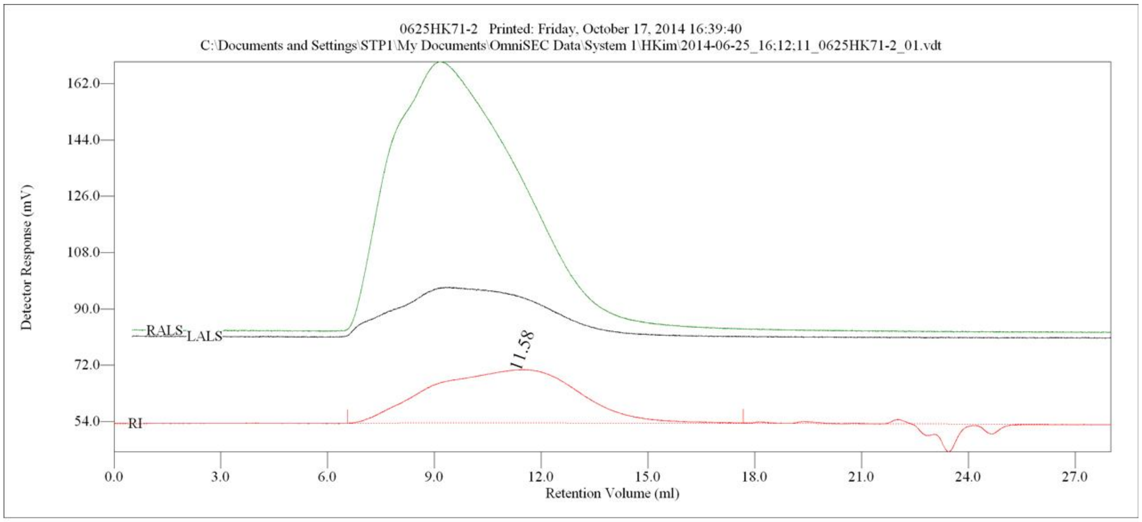

Molecular weight data for peak 1 is non-monotonic. Please read Help - FAQ entry.

Multi-Detectors - Homopolymers : Results

Peak RV - (ml)	11.580
Mn - (Daltons)	162,611
Mw - (Daltons)	237,137
Mz - (Daltons)	339,129
Mp - (Daltons)	146,296
Mw / Mn	1.458
Percent Above Mw: 0	0.000
Percent Below Mw: 0	5.900
IV - (dl/g)	0.0000
Rh - (nm)	0.000
Rg - (nm)	No Calc
Wt Fr (Peak)	1.000
Mark-Houwink a	0.000
Mark-Houwink logK	0.000
Branches	0.000
Branch Freq.	0.000
RI Area - (mvml)	83.07
UV Area - (mvml)	0.00
RALS Area - (mvml)	359.42
LALS Area - (mvml)	74.16
IVDP Area - (mvml)	0.00

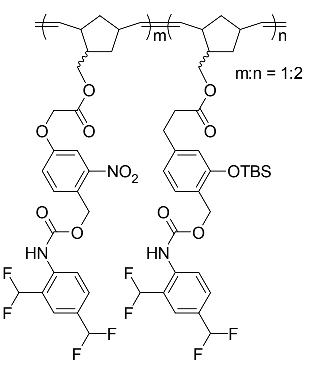

1

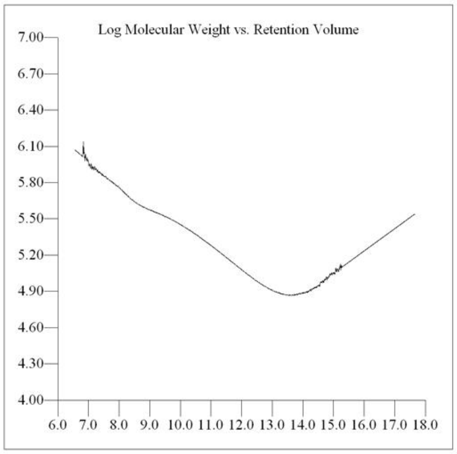

Sample Parameters	Input	Calculated
Sample Conc - (mg/ml)	6.600	0.000
Sample Recovery (%)	0.000	64.993
dn/dc - (ml/g)	0.1850	0.0000
dA/dc - (ml/g)	1.0000	0.0000

Annotation	
Method File	080314CALIBRATION-0000.vcm
Limits File	0625HK71-2_01-Calibration 10-22-13-0005-0003.lim
Date Acquired	Jun 25, 2014 - 16:12:11
Solvent	DMF
Acquisition Operator	admin : Administrator
Calculation Operator	admin : Administrator
Column Set	Varian
System	System 1
Flow Rate - (ml/min)	1.000
Inj Volume - (ul)	50.0
Volume Increment - (ml)	0.00333
Detector Temp. - (deg C)	22.0
Column Temp. - (deg C)	25.0
OmniSEC Build Number	359

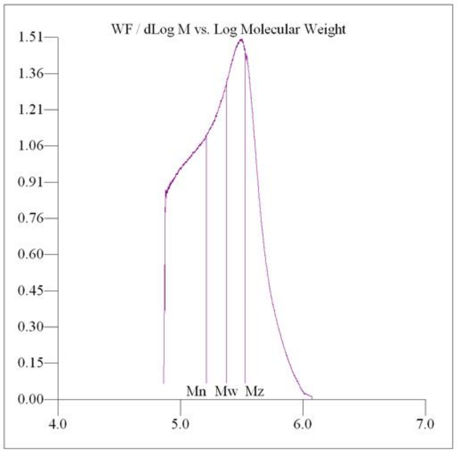

Figure S31. GPC chromatogram of copolymer **1** (M_n = 160 kDa).

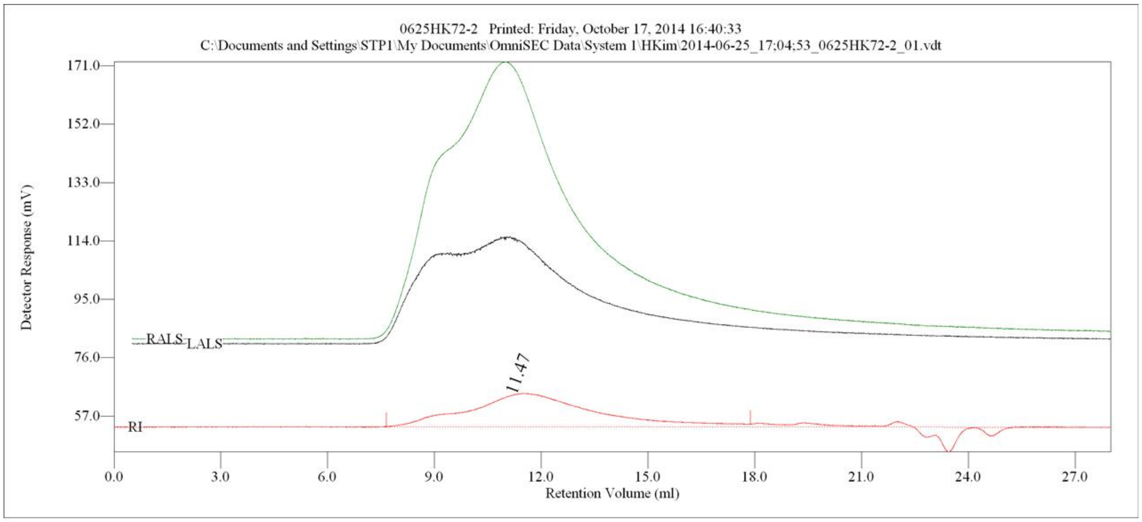

Molecular weight data for peak 1 is non-monotonic. Please read Help - FAQ entry.

Multi-Detectors - Homopolymers : Results

Peak RV - (ml)	11.473
Mn - (Daltons)	287,484
Mw - (Daltons)	334,377
Mz - (Daltons)	420,093
Mp - (Daltons)	260,229
Mw / Mn	1.163
Percent Above Mw: 0	0.000
Percent Below Mw: 0	32.428
IV - (dl/g)	0.0000
Rh - (nm)	0.000
Rg - (nm)	No Calc
Wt Fr (Peak)	1.000
Mark-Houwink a	0.000
Mark-Houwink logK	0.000
Branches	0.000
Branch Freq.	0.000
RI Area - (mvml)	44.62
UV Area - (mvml)	0.00
RALS Area - (mvml)	375.16
LALS Area - (mvml)	168.71
IVDP Area - (mvml)	0.00

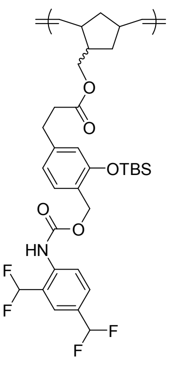

6

Sample Parameters	Input	Calculated
Sample Conc - (mg/ml)	5.600	0.000
Sample Recovery (%)	0.000	41.140
dn/dc - (ml/g)	0.1850	0.0000
dA/dc - (ml/g)	1.0000	0.0000

Annotation	
Method File	080314CALIBRATION-0000.vcm
Limits File	_0625HK72-2_01-Calibration 10-22-13-0005-0002.lim
Date Acquired	Jun 25, 2014 - 17:04:53
Solvent	DMF
Acquisition Operator	admin : Administrator
Calculation Operator	admin : Administrator
Column Set	Varian
System	System 1
Flow Rate - (ml/min)	1.000
Inj Volume - (ul)	50.0
Volume Increment - (ml)	0.00333
Detector Temp. - (deg C)	22.0
Column Temp. - (deg C)	25.0
OmniSEC Build Number	359

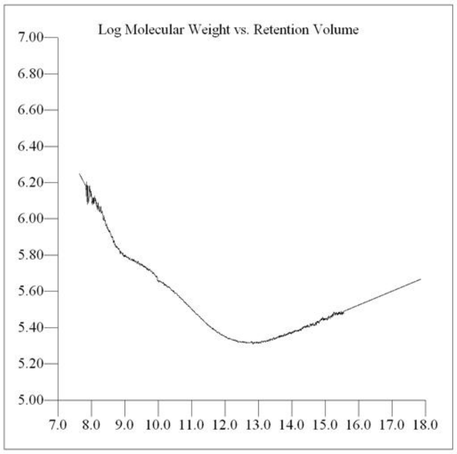

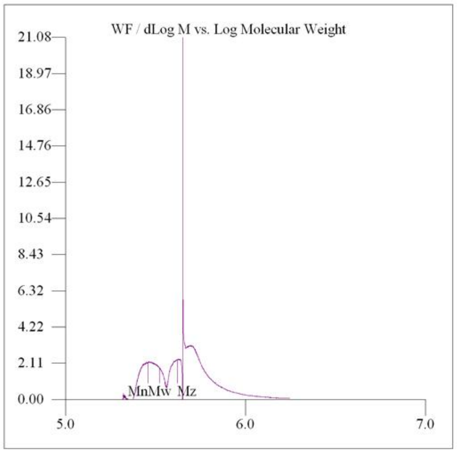

Figure S32. GPC chromatogram of homopolymer **6** (M_n = 290 kDa).

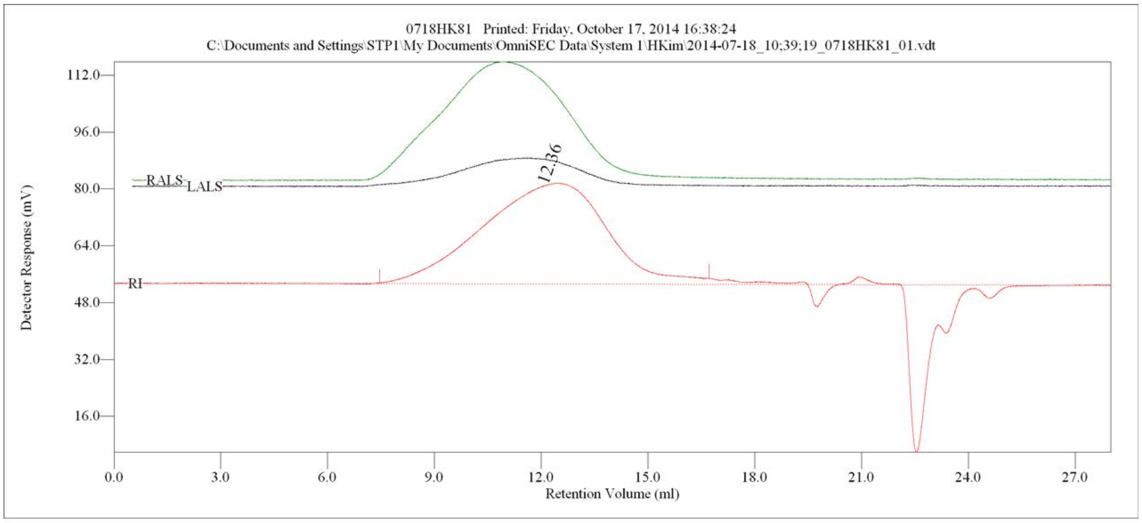

Molecular weight data for peak 1 is non-monotonic. Please read Help - FAQ entry.

Multi-Detectors - Homopolymers : Results

Peak RV - (ml)	12.360
Mn - (Daltons)	73,139
Mw - (Daltons)	114,382
Mz - (Daltons)	167,517
Mp - (Daltons)	84,277
Mw / Mn	1.564
Percent Above Mw: 0	0.000
Percent Below Mw: 0	0.000
IV - (dl/g)	0.0000
Rh - (nm)	0.000
Rg - (nm)	No Calc
Wt Fr (Peak)	1.000
Mark-Houwink a	0.000
Mark-Houwink logK	0.000
Branches	0.000
Branch Freq.	0.000
RI Area - (mvml)	114.13
UV Area - (mvml)	0.00
RALS Area - (mvml)	133.72
LALS Area - (mvml)	29.78
IVDP Area - (mvml)	0.00

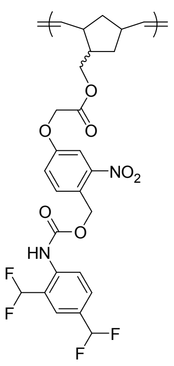

7

Sample Parameters	Input	Calculated
Sample Conc - (mg/ml)	5.000	0.000
Sample Recovery (%)	0.000	117.867
dn/dc - (ml/g)	0.1850	0.0000
dA/dc - (ml/g)	1.0000	0.0000

Annotation	
Method File	080314CALIBRATION-0000.vcm
Limits File	0718HK81_01-080314CALIBRATION-0000-0000.lim
Date Acquired	Jul 18, 2014 - 10:39:19
Solvent	DMF
Acquisition Operator	admin : Administrator
Calculation Operator	admin : Administrator
Column Set	Varian
System	System 1
Flow Rate - (ml/min)	1.000
Inj Volume - (ul)	50.0
Volume Increment - (ml)	0.00333
Detector Temp. - (deg C)	22.0
Column Temp. - (deg C)	25.0
OmniSEC Build Number	359

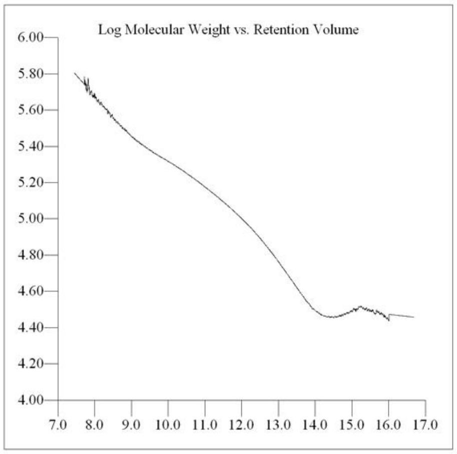

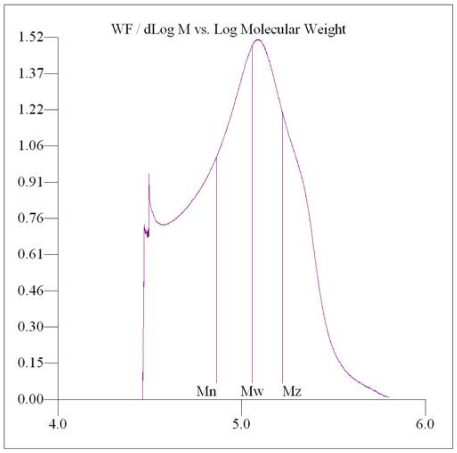

Figure S33. GPC chromatogram of homopolymer **7** (M_n = 73 kDa).
